# Preoperative planning and tracheal stent design in thoracic surgery: a primer for the 2017 Radiological Society of North America (RSNA) hands-on course in 3D printing

**DOI:** 10.1186/s41205-017-0022-3

**Published:** 2017-12-06

**Authors:** Leonid Chepelev, Carolina Souza, Waleed Althobaity, Olivier Miguel, Satheesh Krishna, Ekin Akyuz, Taryn Hodgdon, Carlos Torres, Nicole Wake, Amy Alexander, Elizabeth George, Anji Tang, Peter Liacouras, Jane Matsumoto, Jonathan Morris, Andy Christensen, Dimitrios Mitsouras, Frank Rybicki, Adnan Sheikh

**Affiliations:** 1 Department of Medical Imaging, The Ottawa Hospital, University of Ottawa School of Medicine, Ottawa, ON Canada; 2 Department of Radiology, New York University, New York, NY USA; 3 Department of Radiology, Mayo Clinic, Rochester, MN USA; 4 Department of Radiology, Brigham and Women’s Hospital, Boston, MA USA; 5 Department of Radiology, Walter Reed National Military Medical Center, Bethesda, MD USA; 6 SOMADEN LLC, Littleton, CO USA

**Keywords:** 3D printing, Pancoast tumor, Tracheal Stenosis, Cancer, Segmentation, Computer-aided design, Thoracic surgery, Implant, Radiological Society of North America, Precision medicine

## Abstract

In this work, we provide specific clinical examples to demonstrate basic practical techniques involved in image segmentation, computer-aided design, and 3D printing. A step-by-step approach using United States Food and Drug Administration cleared software is provided to enhance surgical intervention in a patient with a complex superior sulcus tumor. Furthermore, patient-specific device creation is demonstrated using dedicated computer-aided design software. Relevant anatomy for these tasks is obtained from CT Digital Imaging and Communications in Medicine images, leading to the generation of 3D printable files and delivery of these files to a 3D printer.

**Electronic supplementary material:**

The online version of this article (10.1186/s41205-017-0022-3) contains supplementary material, which is available to authorized users.

## Introduction

### General overview

**Fig. 1:**
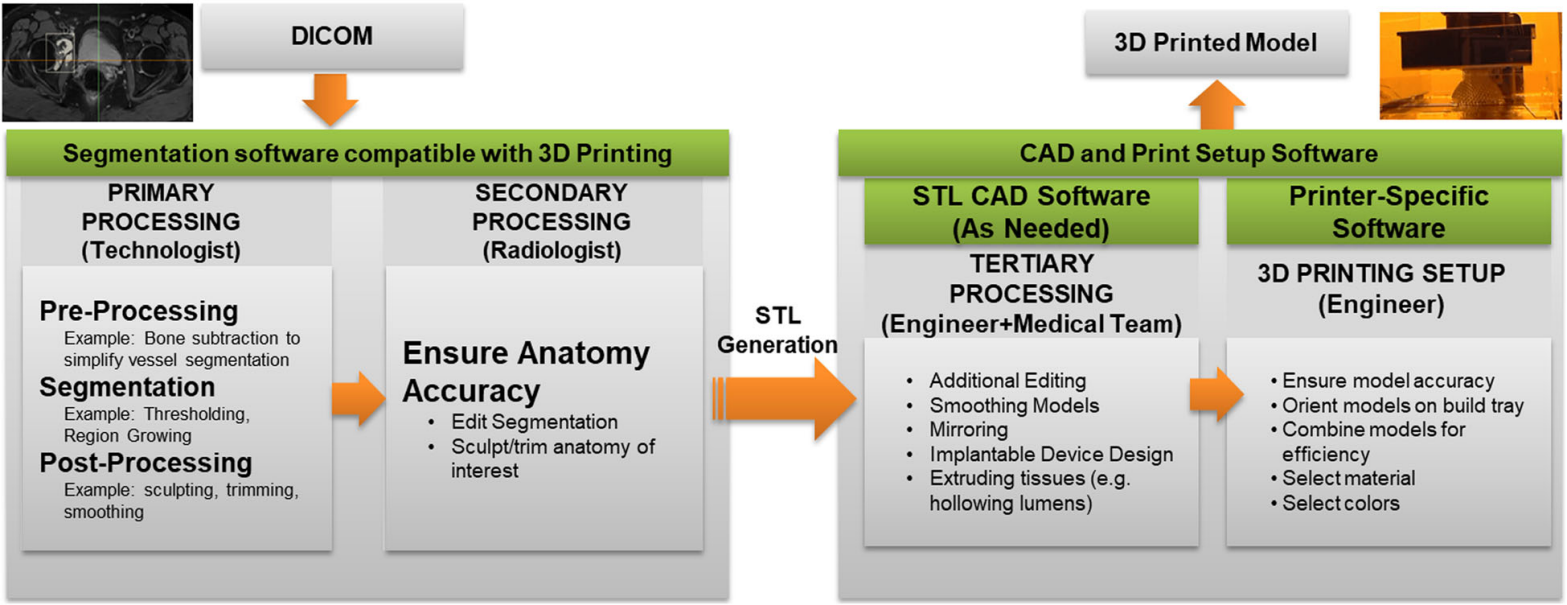
General 3D Printing process overview, adapted from Mitsouras et al. [3]

This is the third publication in an educational series presented by our group for hands-on 3D printing teaching at the annual meeting of the Radiological Society of North America (RSNA) [1, 2]. The overall 3D printing workflow and the involved software and hardware technologies (Fig. 1) have been reviewed at length [3]. In basic terms, clinical 3D printing begins with the acquisition of cross-sectional images. Among cross-sectional techniques, Computed Tomography (CT) is the most common volumetric imaging modality [4]. The ubiquity, high signal-to-noise ratio, contrast enhancement options, and spatial resolution account for the popularity of this modality in 3D printing. It is preferable to segment images acquired with isotropic voxels, with side length on the order of 1 mm. Intravenous contrast should be timed to optimize visualization of the relevant pathology and anatomy.

Segmentation can be regarded as the process of separation of the relevant anatomy to be included within the 3D printed model. From a technical perspective, segmentation defines the criteria for the voxels to be included in the 3D printed model. For example, such criteria may include connectivity to a seed point in the volumetric data selected by a user. Another example involves the selection of all the voxels with a corresponding density value within a specified range of Hounsfield Units (HU) for Computed Tomography (CT) data or intensity values for Magnetic Resonance Imaging (MRI) data depending on the specific application and suitability of the imaging modality for the demonstration of the pathology in question. In yet another example, all the voxels within a specific manually defined geometric bounding box may be selected. The sequential application of and combinations of such selection criteria using manual or semi-automated techniques may be employed in segmentation, resulting in the selection of the desired anatomy.

The process of 3D visualization – defined as the collection of methods and technologies used to visualize the 3D representation of cross-sectional data volumes on two-dimensional computer screens - typically ends at isolating and displaying a set of segmented voxels from Digital Imaging and Communications in Medicine (DICOM) images. Additional manipulations are required to enable 3D printing. Specifically, the collection of voxels representing a desired anatomy needs to be transformed into a printable 3D object via a process referred to as tessellation.

Tessellation is widely used in computer graphics to approximate shapes using a set of triangles. The more triangles used in tessellation, the more refined a shape becomes. Unfortunately, the transformation of a segmented Region of Interest into a 3D object using tessellation does not ensure model printability or stability. For instance, it may be necessary for the model to be further smoothed, non-printable parts may be removed or mathematically adjusted, and vulnerable areas may be reinforced using a range of manual manipulations and automated algorithms to ensure models that are robust and to minimize printing failure rates. The result of these operations is stored as an alternative file type, usually a Standard Tessellation Language/Stereolithrography (STL) file submitted to, and recognized by, a 3D printer to build the model.

Once an accurate representation of the relevant patient anatomy is obtained in the form of a printable 3D model, further manipulations using Computer-Aided Design (CAD) software is typically necessary for model refinement or patient-specific instrument design.

### Software overview

To illustrate the most common techniques in using segmentation and post-processing software, this work uses Materialise Mimics inPrint and 3-matic (Materialise, Leuven, Belgium). Although numerous software packages are available to carry out these tasks, the software presented in this work is US FDA-cleared for the medical applications demonstrated in this work. Mimics inPrint software facilitates the processing of 2D image data acquired from axial imaging (CT, MRI) to create 3D printable models. While a significant portion of Materialise Mimics functionality is present in this package, certain higher-order operations, including various segmentation algorithms present in more advanced packages such as Materialise Mimics, are omitted in this paper for simplicity.

Materialise 3-matic Medical software contains a set of CAD tools useful in the design of medically relevant models. This software enables the manipulation of patient-derived 3D models as well as creation and design of entirely new models in the context of patient anatomy. The application of this package in anatomical reconstruction and medical device creation will be explored in this work.

We also discuss the use of build preparation software, namely GrabCAD (Stratasys Ltd., MN, USA). While the build preparation is highly variable, and is usually bundled with the specific printer used, the GrabCAD Print software and services demonstrate the typical features of such software in a single package. GrabCAD is an online 3D printed model preparation, management, and monitoring service and software. GrabCAD enables the preparation of 3D models for printing and enables the exploration of factors such as part placement, material costs, build times, and material selection to optimize the 3D printing process. The built-in adaptable software interface allows the preparation of prints using complex multi-material PolyJet technology and simpler FDM-based printers alike.

### Clinical overview

Superior sulcus (Pancoast) tumors frequently involve the pleura, bones, vessels, and nerves, and therefore present challenging management decisions that can be aided by 3D printing [5, 6]. Specifically, the choice of whether to pursue surgical management can be swayed by a physical, 3D printed model. Depending on the extent of disease, and following neoadjuvant radiotherapy, surgical management may involve minimally invasive surgery and is often quite delicate, requiring excellent visualization and understanding of the involved anatomy to optimize patient outcomes. Reconstruction of the chest wall is typically not performed in the cases that involve only the first three ribs or where the chest wall defect is entirely covered by the scapula. Cases outside of this domain are typically repaired using a Gore-Tex patch. While cases involving vertebral reconstruction and spinal fusion have been demonstrated, such cases are relatively rare. In cases where neoplastic involvement results in sufficient mass effect to induce significant tracheal or bronchial stenosis, stents may be considered to improve patient quality of life. The purpose of this work is to explore the basic approaches that may be used in complex neoplastic pathology model creation and patient-specific tracheal stent design used in medical 3D printing. This will be accomplished starting with arterial-phase axial CT images using 1 mm reconstruction in soft tissue windows. The readers will be exposed to a broad range of basic approaches that may be used in creating a complex neoplastic pathology model and the digital design process of creating a tracheal stent for 3D printing.

## Segmentation

**Fig. 2:**
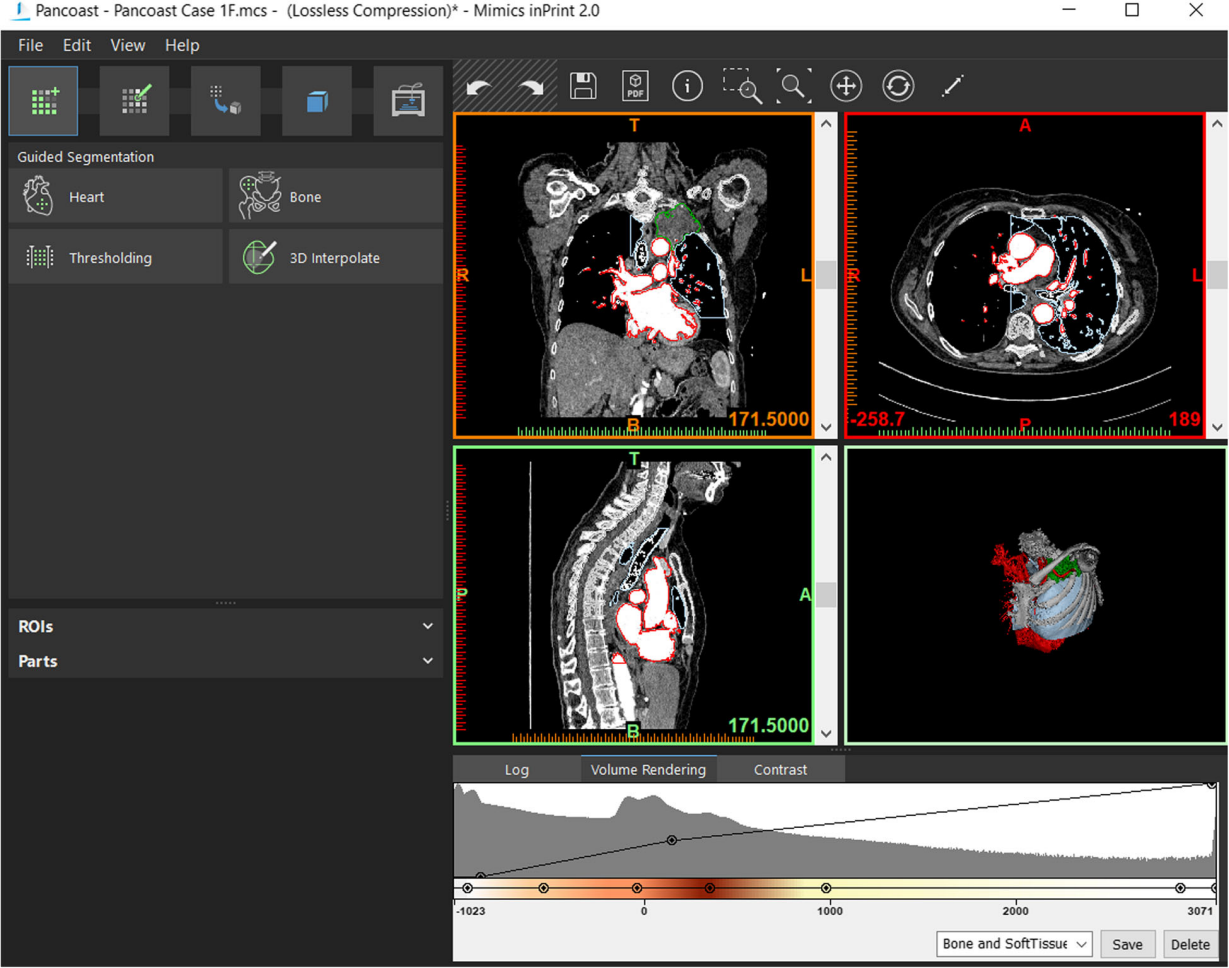
Layout of the inPrint software graphical user interface. On the top left, the menu toolbar with File, Edit, View, and Help menus. Below this, menu toolbar with segmentation and model editing menus. The operation toolbar (titled Guided Segmentation on this view) contains the available operations and operation settings. Below this, the ROI and Part lists can be found. Top right, the three orthogonal planes and the 3D visualizations are shown. Finally, the lower right is reserved for software logs and various HU distribution and window/level visualizations

Segmentation separates the voxels of interest using a range of properties, such as their HU values, presence within a specific predefined bounding box ranging from the entire study volume to a limited region of interest, or a degree of contiguity to define a single continuous structure such as a rib or a vessel. The files included with this work in supporting documentation can be opened by double-clicking after the installation of the inPrint software, or from within the inPrint software using the File menu on systems where this software is installed. The main program window contains the toolbox on the left as well as project visualization with the three orthogonal visualization planes and the 3D rendering window (Fig. 2). DICOM images may be imported and loaded into a project file, using a similar intuitive wizard from the File menu as well. Before progressing further, please take a moment to familiarize yourself with the software shortcuts and anatomy (Table 1).

**Table 1:** Selected software shortcuts in the in Print software

Shortcut	Action
Scroll wheel (center mouse) OR Shift right click + drag	Pan: Move the mouse while keeping the center click pressed
Right click + drag (2D Views)	Zoom: Move the mouse vertically while keeping the button pressed
CTRL + right click + drag (3D View)	Zoom: Move the mouse vertically while keeping the buttons pressed
Arrow Up/Scroll wheel up	Go to next slice
Arrow Down/Scroll wheel down	Go to previous slice
Page Up	Skip 10 slices upward
Page Down	Skip 10 slices downward
CTRL + L	Make slice indicators visible/invisible
SPACE	Zoom the chosen view to full screen and back
Backspace	Switch between two window states
CTRL + Z	Undo the previous action.
CTRL + Right click + Drag (2D Views)	Adjusts window/level in 2D images

### Segmentation of bones

**Fig. 3:**
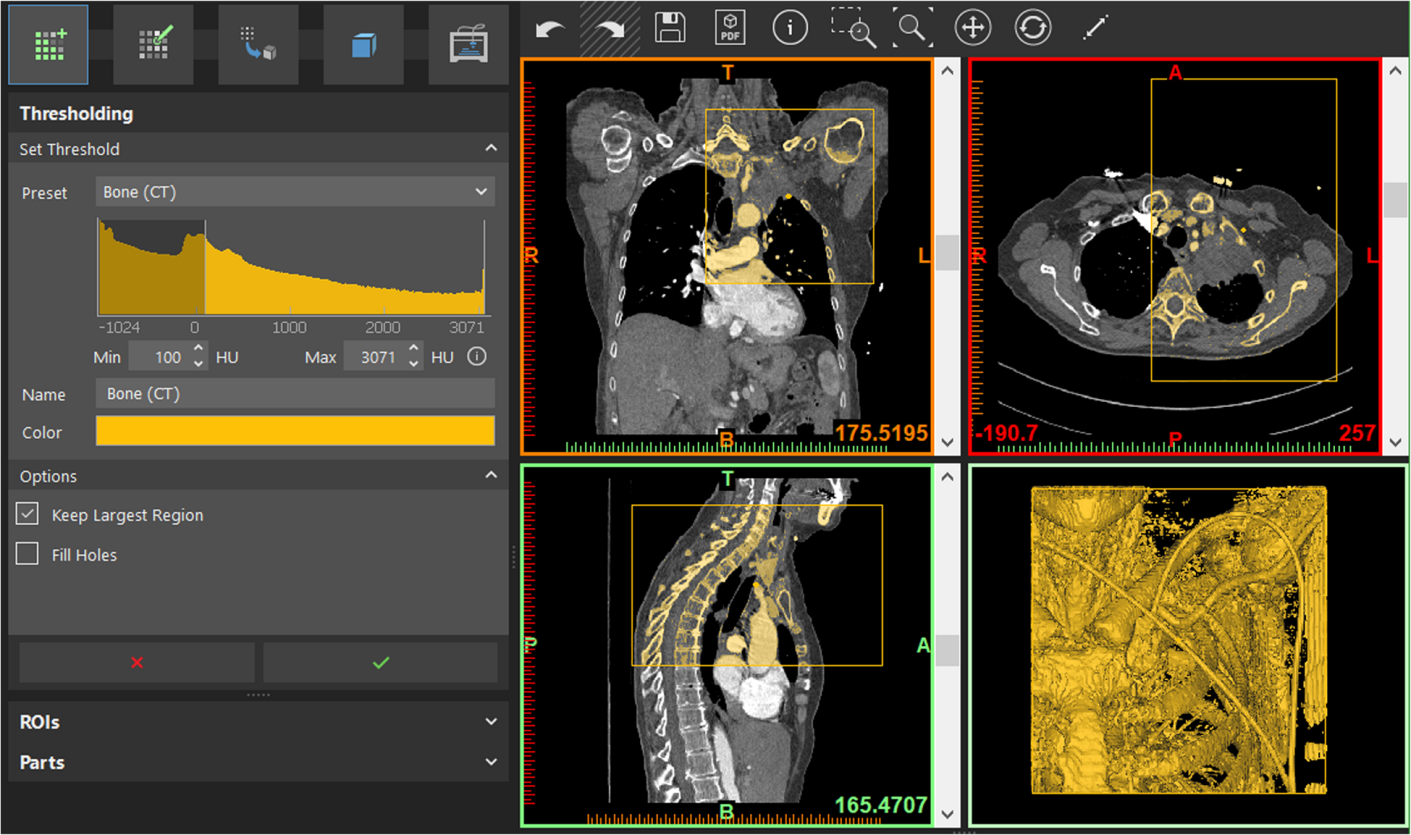
Setting up the segmentation for osseous structures

We begin this segmentation step by identifying the thoracic skeletal structures. To do this, we will use Thresholding tool from the Create ROI menu and select the Bone (CT) preset to identify a Hounsfield Unit (HU) range that typically corresponds to bones in a typical CT scan. Since this range is not satisfactory in these particular images due to the reconstruction algorithms and acquisition parameters used, we shall adjust the lower HU bounds to 100 by typing this number in the appropriate field (Fig. 3).

We will also adjust the bounding box to exclude the CT table from the ROI by left-clicking and dragging these boundaries, as demonstrated. Since we do not need the lower and the contralateral upper thorax, or the neck, we will adjust the superior and inferior boundaries to solely focus on the tumor, including the acromioclavicular junction and a portion of the shoulder (Fig. 3).

Given this range of HU values, you will notice that the voxels of interest are now highlighted, and are a part of a single preliminary ROI visualized on the 3D view. Although the ROI we are creating occupies a 3D space, and is composed of thousands of individual voxels, it cannot be directly submitted for 3D printing, as it needs to be converted to a three-dimensional model readable by a 3D printer, such as an STL model. As stated earlier, in an STL model, all geometric shapes are redefined as a collection of surface triangles that approximate the shape of the object in question through a process known as “tessellation”. In general, the more triangles are used, the higher is the fidelity of the representation, which could result in greater computational demand for manipulating the object.

To summarize, for the purposes of this discussion, the ROI is a collection of cubic or cuboid voxels different from a 3D printable model. The ROI will be converted to printable file after we ensure that the appropriate voxels are included within the ROI first.

**Fig. 4:**
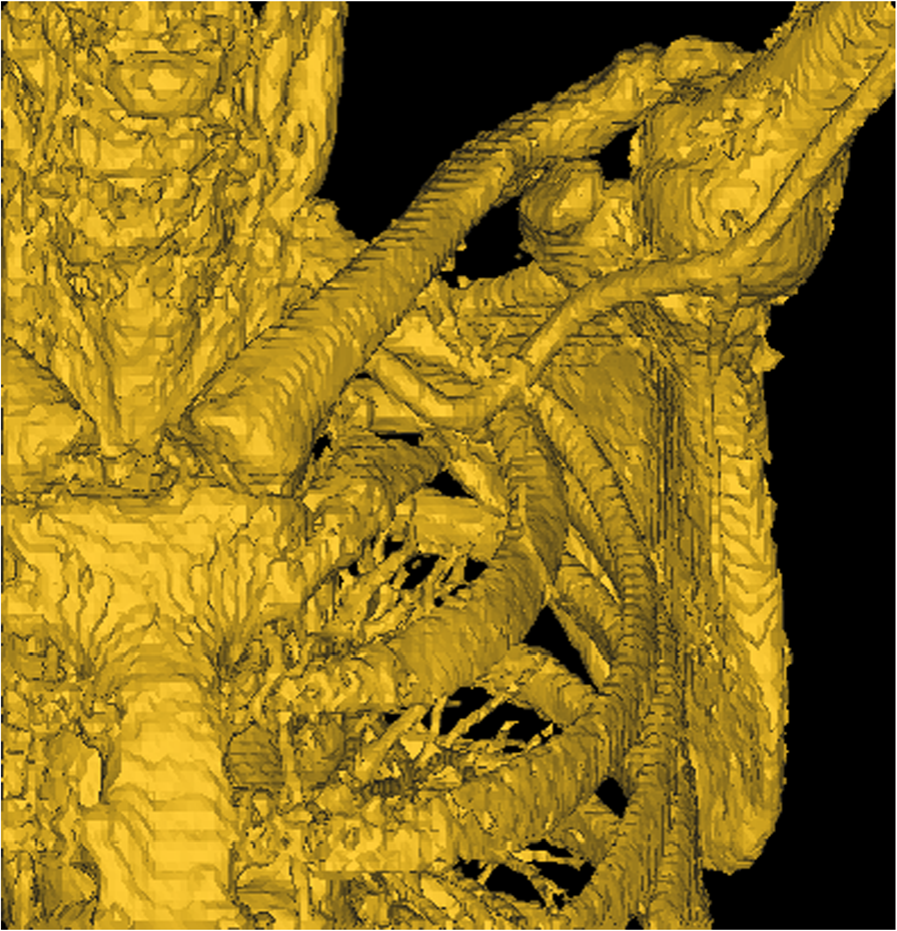
Result of the segmentation operation with HU, contiguity, and bounding box restrictions

Notice that a number of structures have been included within the current preliminary ROI, including the heart and the major vasculature, as well as the leads on the patient’s anterior chest wall. Many of these undesirable voxels will be removed in the later stages of the segmentation. Select the option to ‘Keep Largest Region’ to leave only the contiguous voxels within the newly generated ROI and press the green checkmark. After this step is complete, you will note that the ECG leads from the patient’s anterior chest wall and any non-contiguous voxels selected in the previous step have been removed because they were not attached to the largest contiguous voxel ROI (Fig. 4).

You will also notice that the new ROI, titled ‘Bone (CT)’ has appeared in the list of ROIs, and the menu has changed to the Edit ROI menu, which allows separation of unwanted portions of the ROI and combination of different ROIs together using the ‘Split’ tool. We shall take advantage of this tool to remove the great vessels and the included potion of the heart, while leaving the bones in place.

**Fig. 5:**
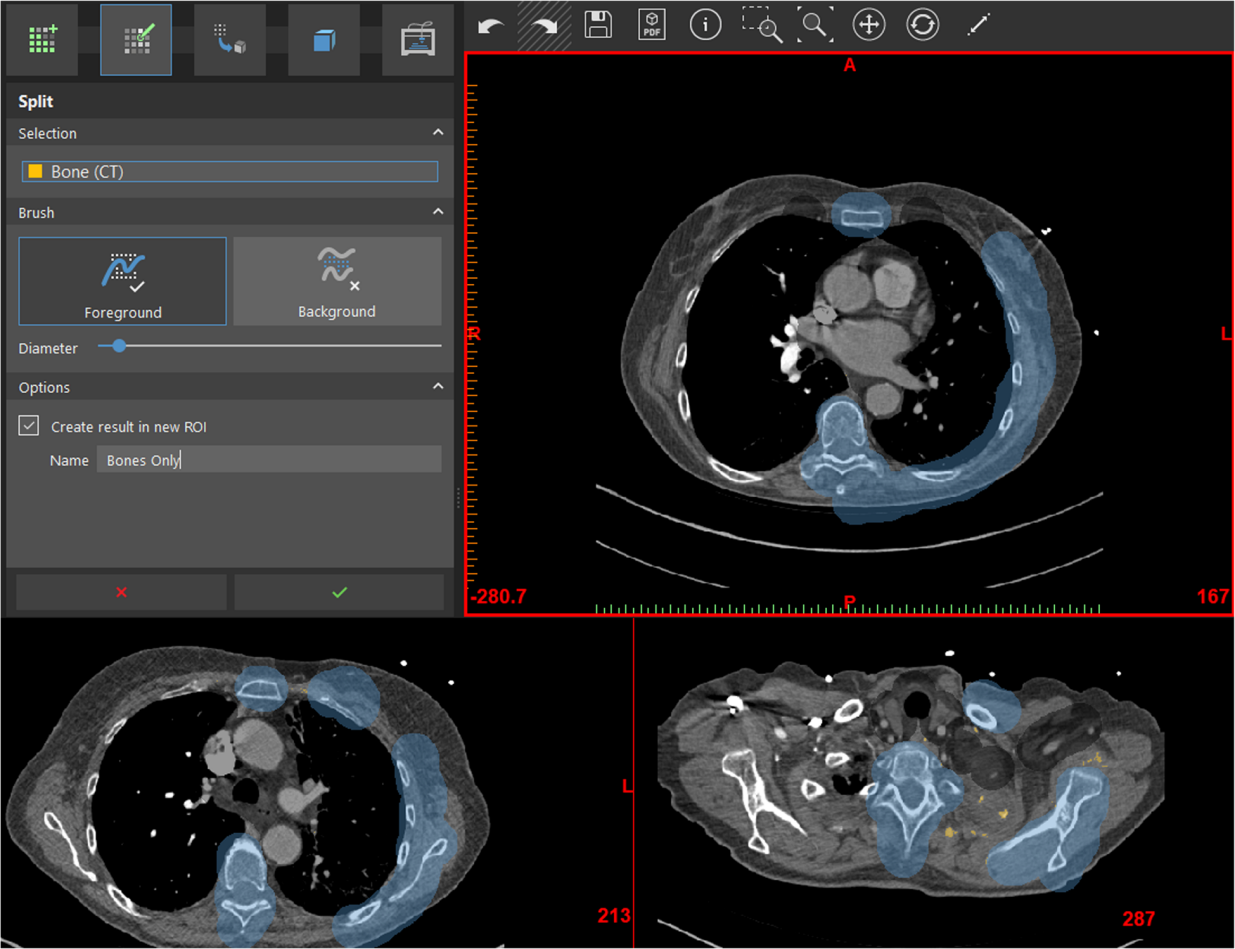
Setting up the splitting tool and marking up the appropriate osseous anatomy as the foreground (blue) and vascular anatomy as background (black). Note image 292 is not shown

To do this, we define the ‘Background’ and the ‘Foreground’ structures on several views using the appropriate brushes within the split tool. We will use the axial view and, clicking the appropriate Background/Foreground buttons, we will outline all bony structures to be included in the Foreground, and everything else as Background on images marked as 167, 213, 287, and 292 as indicated in the lower left corner in the axial view images (Fig. 5). When this is complete, select ‘Create result in new ROI’ option and name it ‘Bones Only’. Press the green checkmark to complete the operation. Now and henceforth, when an operation is set up as indicated, the green checkmark is used in the inPrint software to accept the parameters and execute the operation.

**Fig. 6:**
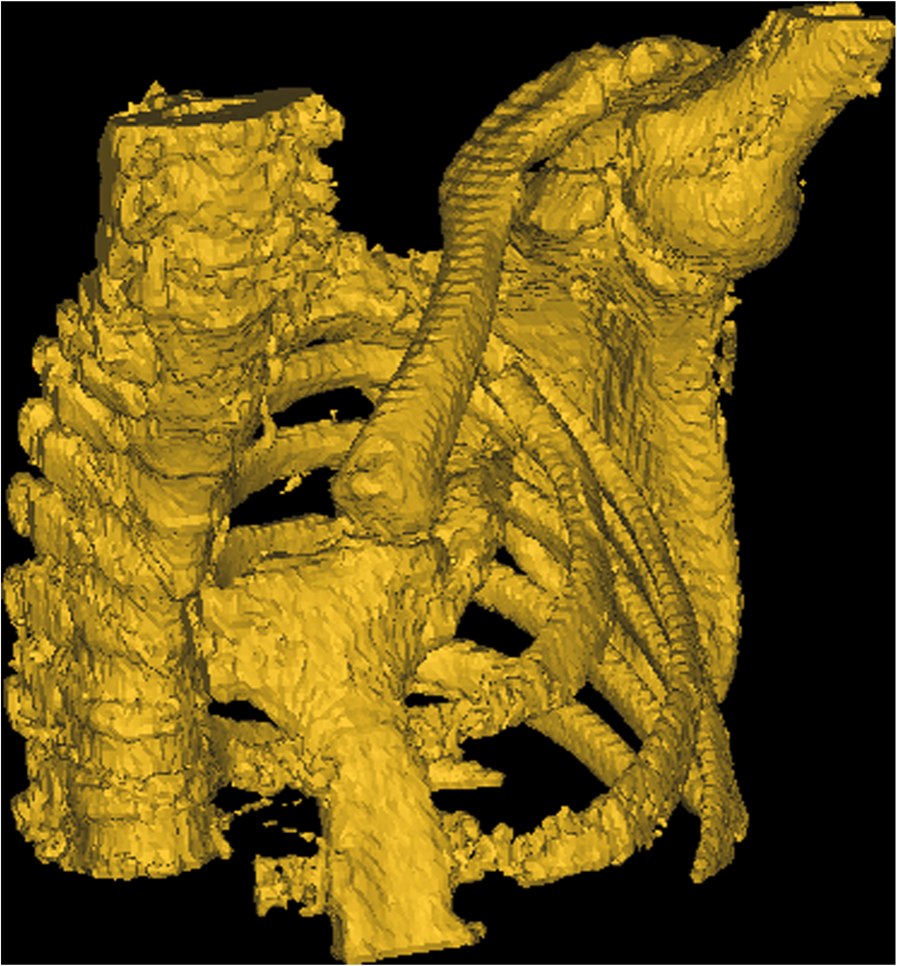
Results of the Split tool, separating the majority of the osseous structures from the vasculature

The resultant osseous structures are well-separated and contain only few residual vessels that can be removed manually (Fig. 6).

**Fig. 7:**
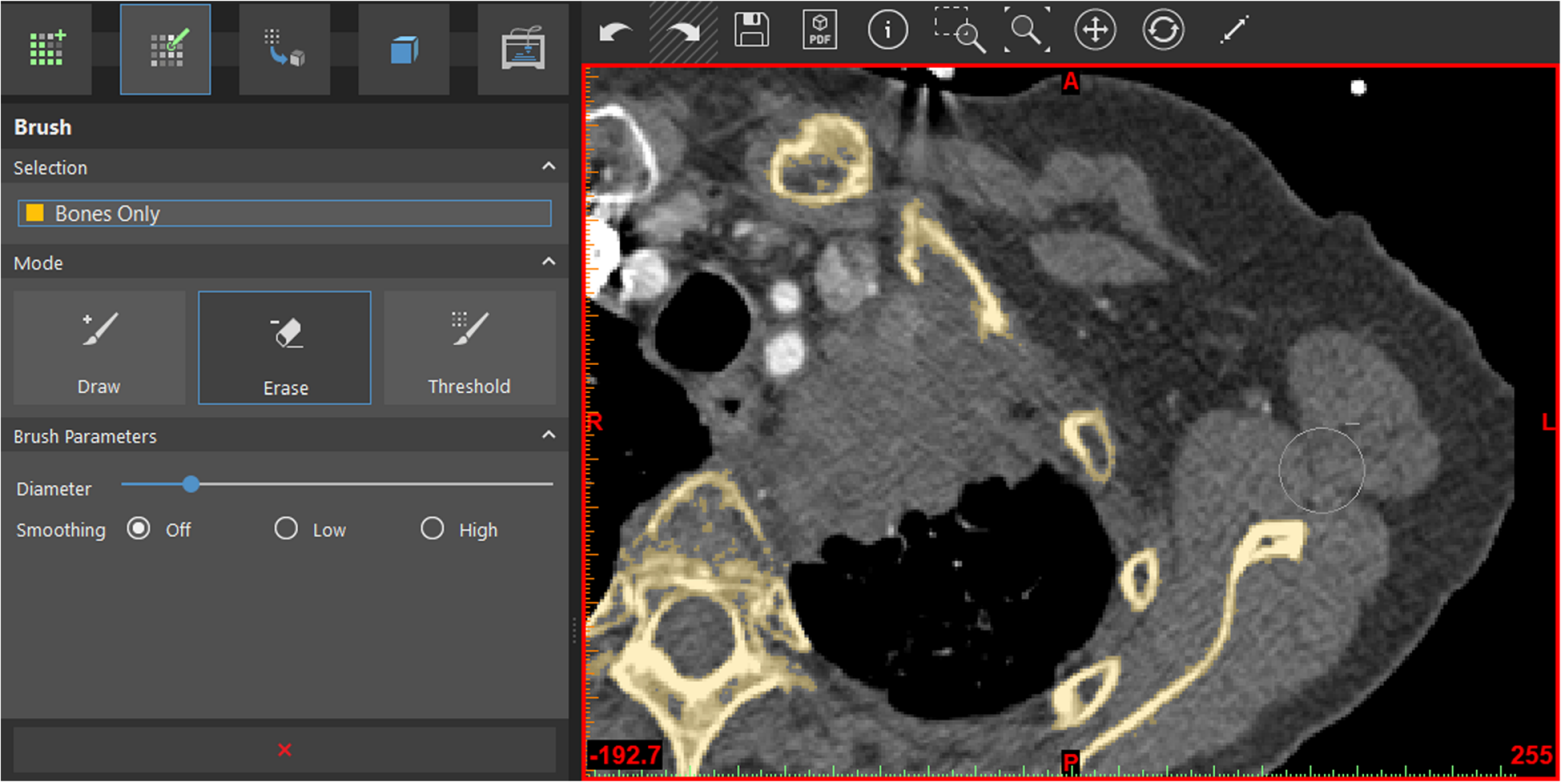
Using the Brush tool to erase unwanted portions of the ROI, slice-by-slice

To touch up the ROI manually, we will select the Brush tool from the ‘Edit ROI’ menu, then the ‘Erase’ brush and on axial images, we will delete the vessel running adjacent to the scapula by editing the ROI on axial images, painting with the ‘Erase’ brush by left-clicking and dragging on the undesired voxels on each relevant axial image (Fig. 7).

**Fig. 8:**
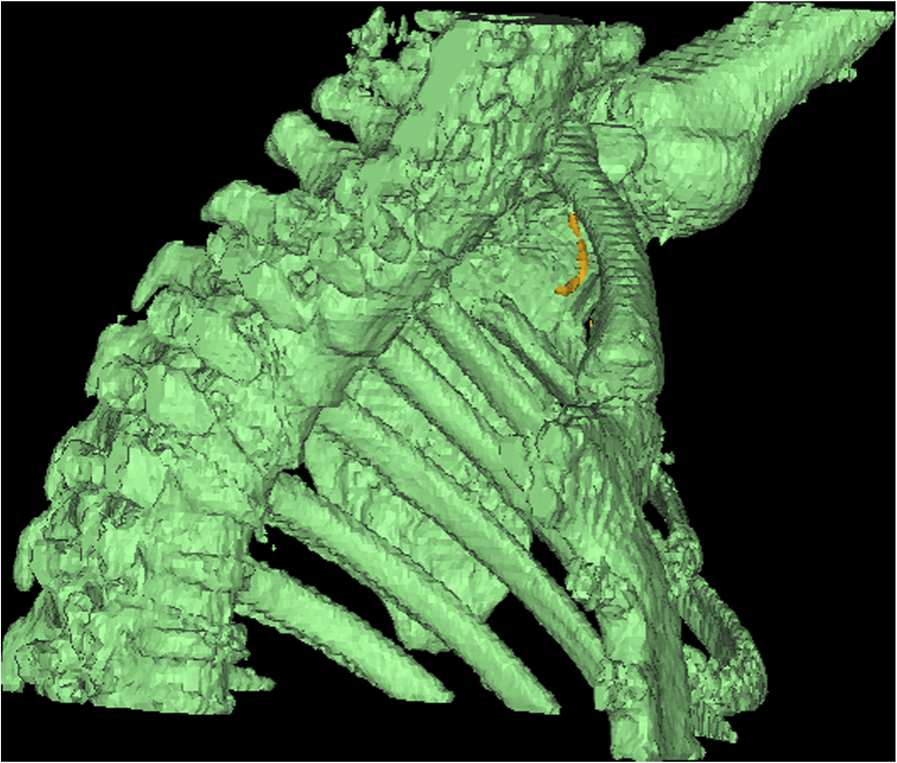
Split tool in action – left clicking on the desired contiguous portion of the anatomy isolates it (green) from the non-contiguous fragments (yellow)

After you are satisfied with your edits, go back to the ‘Edit ROI’ menu and select the ‘Isolate’ tool. This tool allows you to isolate a collection of all contiguous voxels into a single ROI and isolate it from all the non-contiguous, floating parts, which are typically undesirable for the final model. Left-click anywhere on the desired portion of the ROI to see it highlighted green, while the non-connected voxels will remain in their original color (Fig. 8).

**Fig. 9:**
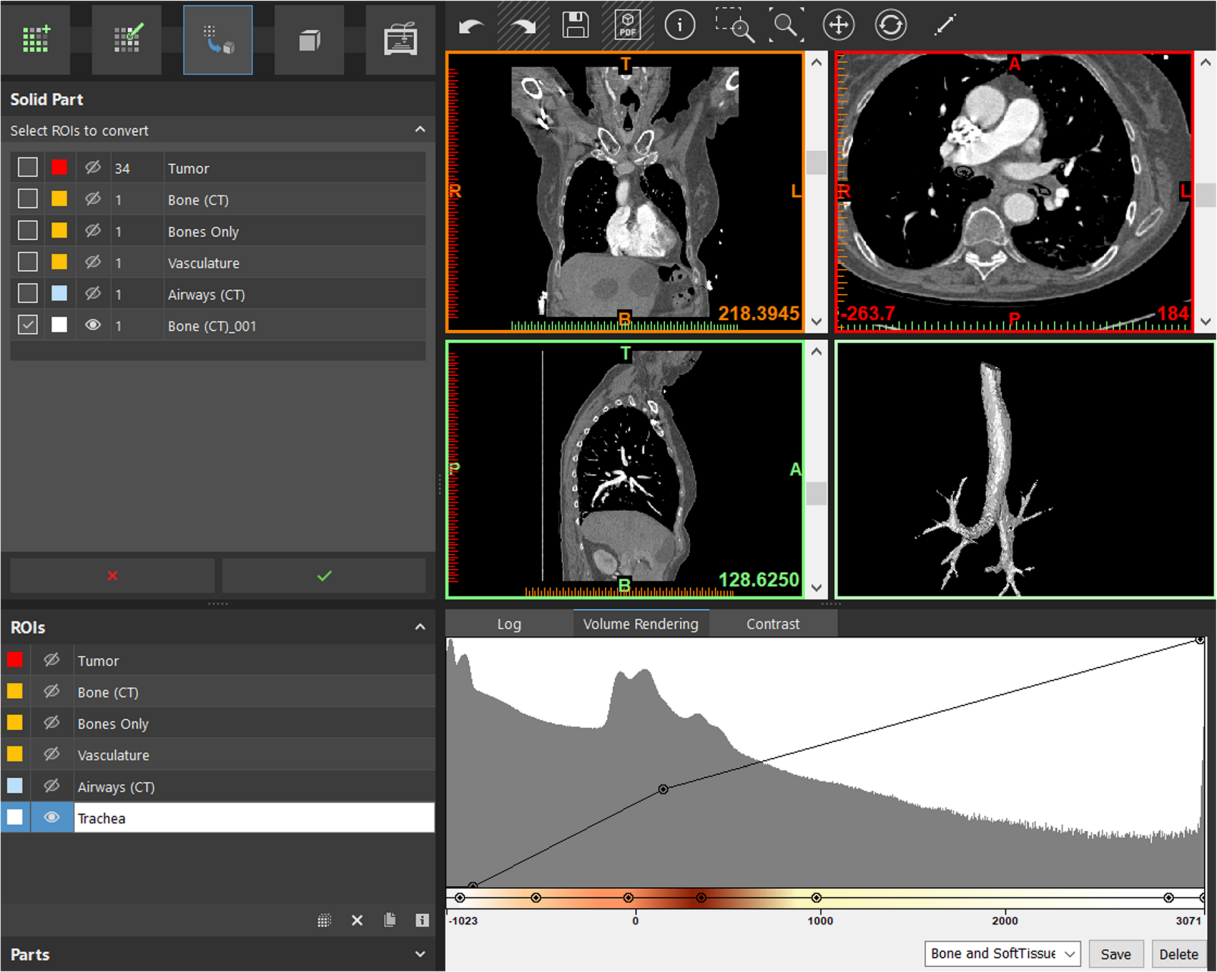
Renaming, showing/hiding, and recoloring ROIs

Note that you can rename the ROIs at any time by single-clicking their names and typing the new name, then pressing ‘Enter’ on the keyboard. Model color can be adjusted to any appealing value from a palette by left-clicking the color square to the left of ROI name (Fig. 9). You can also hide and show a specific ROI by clicking on the eye button next to the ROI name.

### Segmentation of vascular anatomy

While the same process as the one used for the bones can be repeated to segment the major vascular structures, we will use the Subtraction tool from the ‘Edit ROI’ menu since we already have a high-quality ROI that includes bony anatomy that can be subtracted from the ROI that contains the bone and vascular anatomy.

**Fig. 10:**
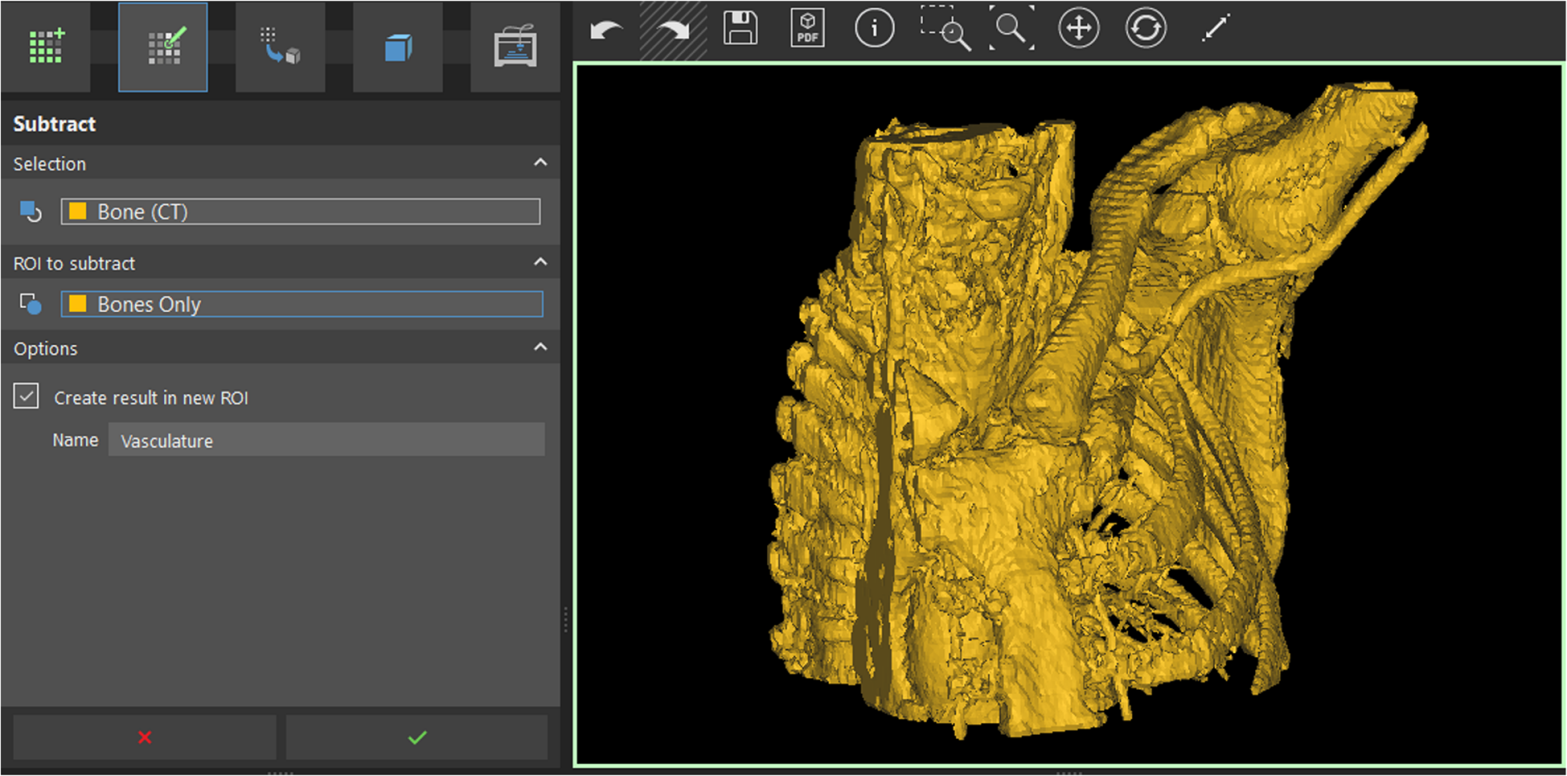
Setting up ROI subtraction

To do this, select the ‘Subtract’ tool from the ‘Edit ROI’ menu. Click on the ‘Selection’ box (here and elsewhere, ‘click’ refers to left-clicking) and then click on the Bone (CT) ROI from the ROI list (Figure). For the ROI to subtract, select the ‘Bones Only’ ROI by the same method (Figure). If you select the wrong ROI, simply click its name on the ‘Selection’ or ‘ROI to subtract’ box and press the ‘Delete’ key on your keyboard, and try again. Select the ‘Create result in new ROI’ option and name it ‘Vasculature’ as shown (Fig. 10). After clicking the green checkbox, this operation will complete and result in vascular anatomy, with inclusion of the thyroid gland, which can be removed later, should this be desired.

**Fig. 11:**
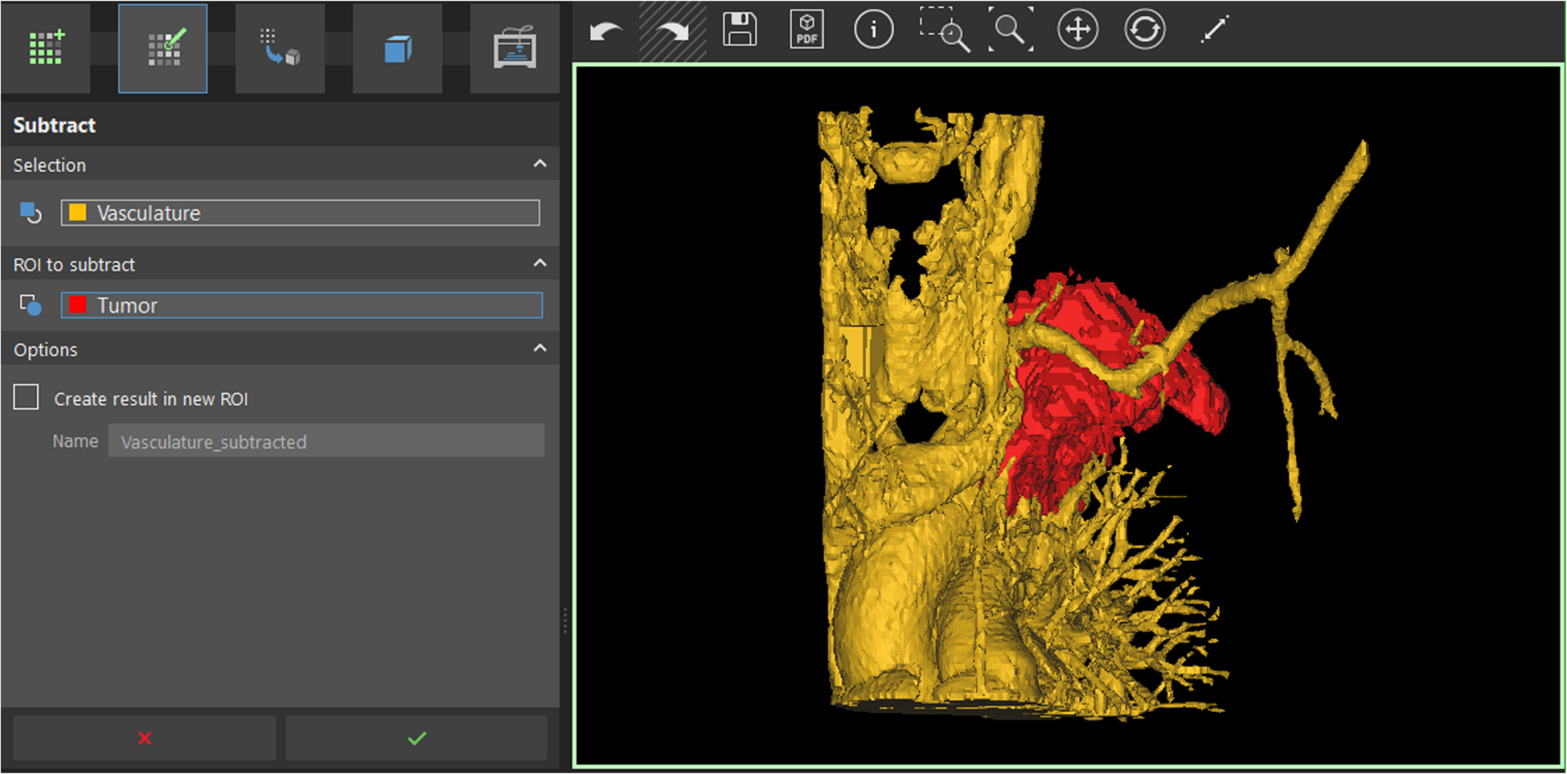
Second application of the ROI subtraction tool.

Notice that portions of soft tissue included in the tumor are present in this model. Use the Subtract tool again to remove the tumor ROI included with the project from the vasculature, using the methodology above. Clean the model up using a combination of the Brush and Isolate tools as discussed earlier (Fig. 11).

Notice that while the arterial anatomy is well-characterized, the veins – a possible significant source of intraoperative hemorrhage – have not been well characterized on this arterial phase study. It may be necessary to either manually reconstruct the venous anatomy using these DICOM images (a time-consuming manual approach using the brush tool on every relevant 2D image), or fuse the available arterial phase ROIs with those obtained from images acquired using a different phase of contrast opacification.

### Segmentation of lungs and major airways

**Fig. 12:**
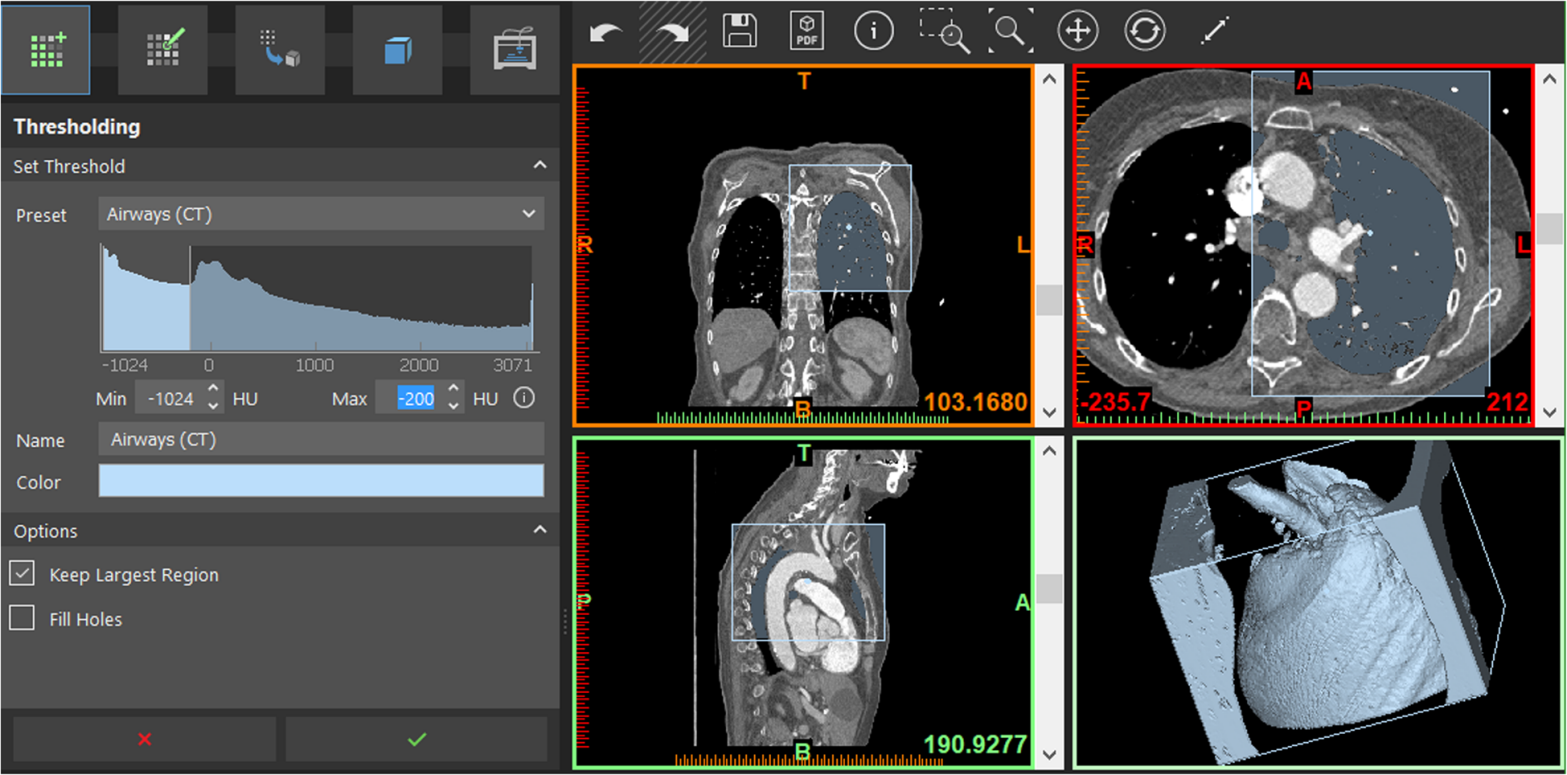
Segmenting the airways using the Airways (CT) preset

In order to segment the lungs and major airways, we will repeat the segmentation from the ‘Create ROI’ menu, use the Thresholding tool and select the ‘Airways (CT)’ preset. Adjust the bounding box to only include the portion of the lungs and airways of interest and select the ‘Keep Largest Region’ option. Select the upper bound of the thresholding operation to be −200 HU before creating the ROI (Fig. 12).

**Fig. 13:**
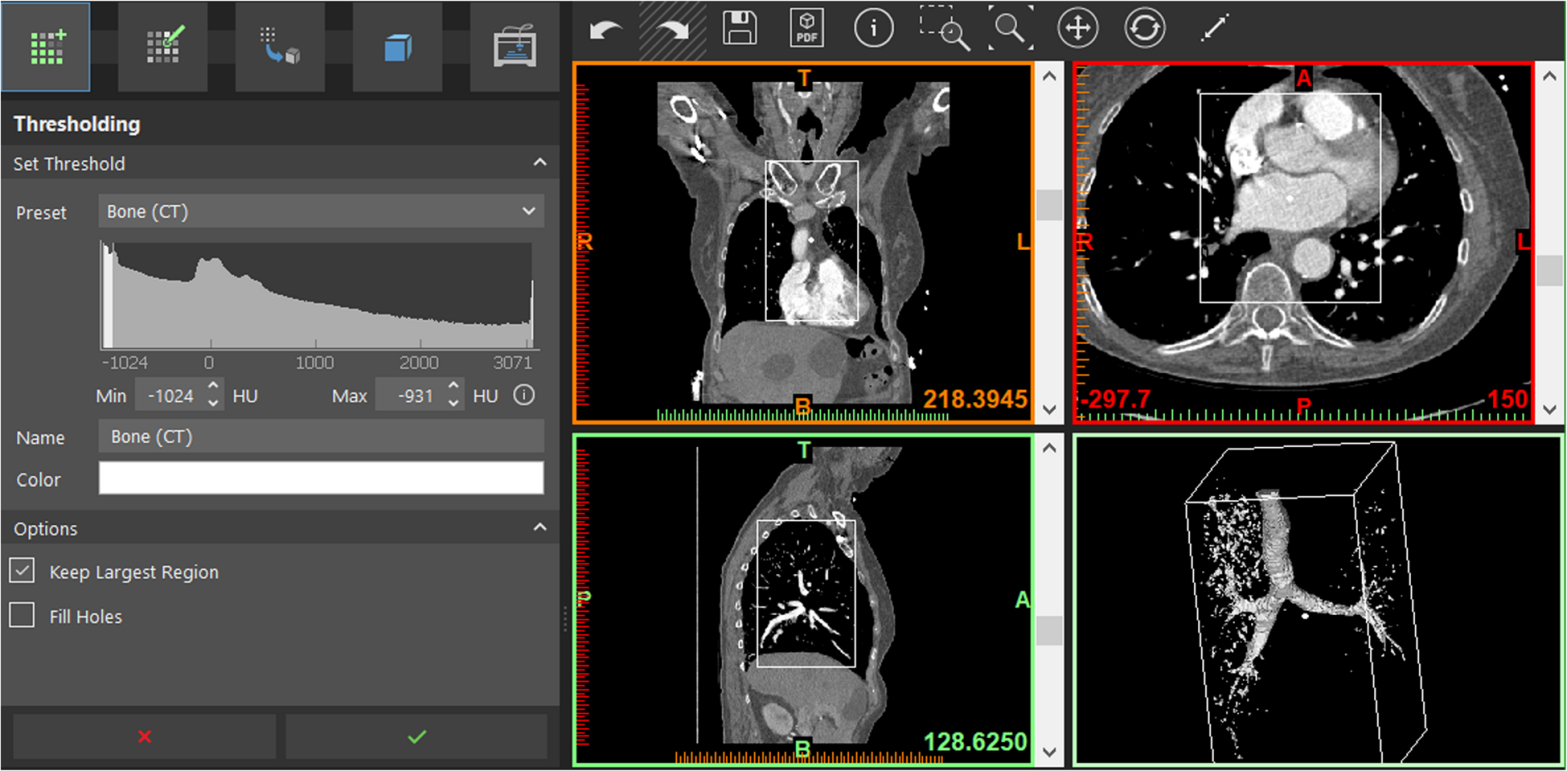
Segmentation of the trachea. Note the HU range and the narrow bounding box adjustments

The result is an ROI that includes air within the lungs and trachea. To better define the intraluminal tracheal configuration, the Thresholding tool can be used again, with −1024 to −931 HU range. Adjust the bounding box to center it on the trachea, and proceed to generate the tracheal ROI by clicking the green checkmark, as indicated (Fig. 13).

### Tumor segmentation

For ease of use, we have included a pre-segmented tumor ROI with the dataset provided. The task of segmenting this ROI from the surrounding soft tissues is time consuming, and the tumor is often difficult to distinguish from the adjacent soft tissues of the chest wall, especially on images obtained from phases where the tumor does not exhibit significant enhancement. The differential enhancement of the tumor in this case is not amenable for semi-automated isolation, necessitating the use of additional intravenous contrast phases, or fusion with other imaging such as MRI or PET where the tumor extent may be more accurately demonstrated, per the discretion of the radiologist and the clinical team. If you wish to segment this tumor manually, you may experiment with thresholding tools to select the soft tissue HU range best depicting the tumor alone to define a starting ROI within the left upper thorax, followed by image-by-image manual edits to segment the tumor.

**Fig. 14:**
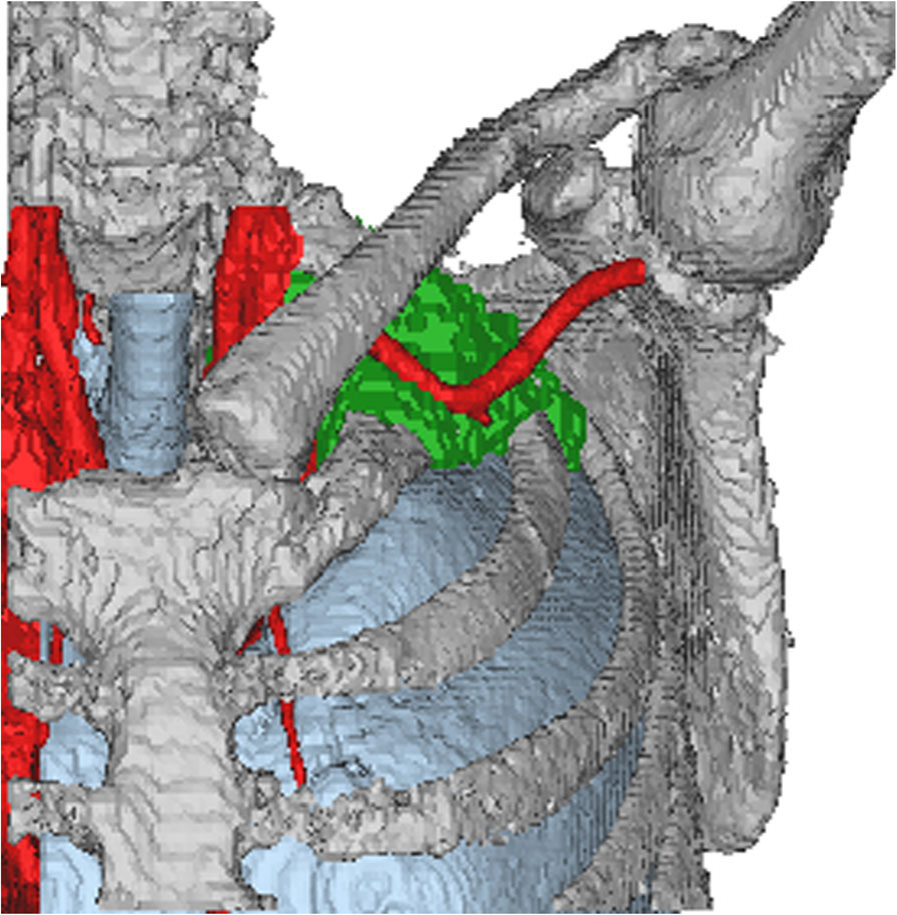
Results of the segmentation

**Fig. 15:**
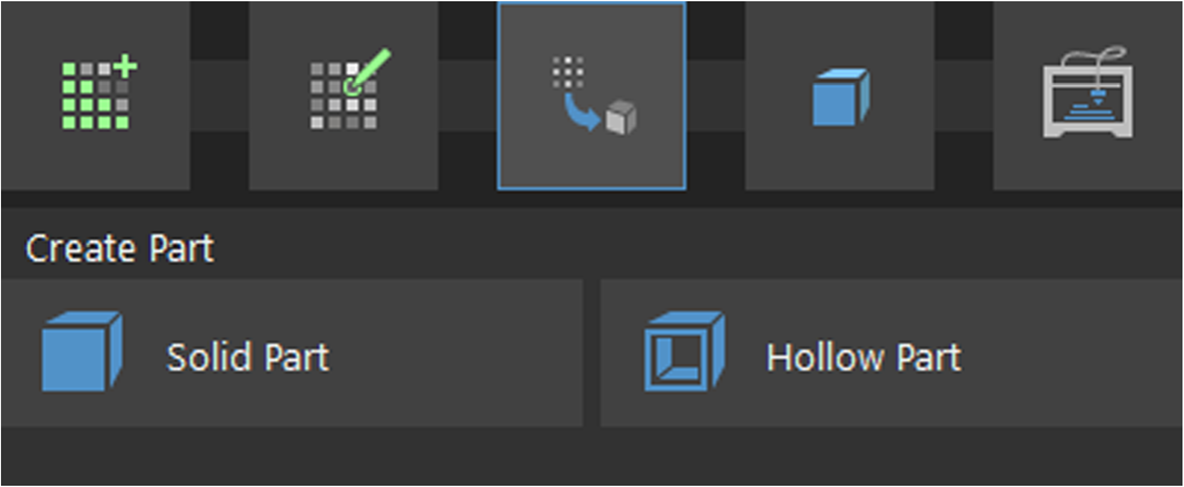
Selection of the Solid Part function from the ‘Create Part’ menu (highlighted)

**Fig. 16:**
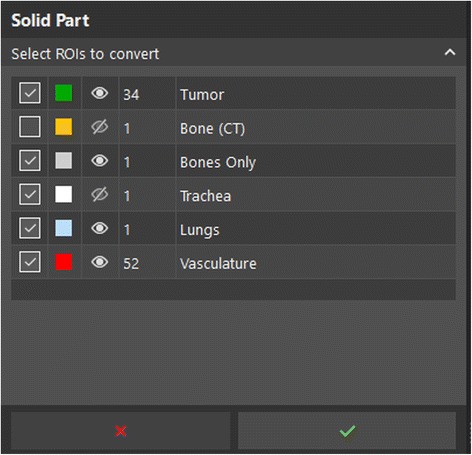
Selection of the ROIs to convert to 3D models

At this time, we have segmented the tumor, vessels, airways, lungs, and bones and are ready for the next step (Fig. 14). Use the ‘Solid Part’ tool from the ‘Create Part’ menu to create 3D models, or parts, for each of the relevant ROI entities to enable further manipulation in CAD software or direct 3D printing (Figs. 15, 16). This constitutes the transition from an ROI composed only of rectangular voxels to 3D models. You will notice some step artifact in the resultant models, relating to the acquisition technique for the original images, not optimized for 3D printing – namely, not highest resolution possible isotropic reconstructions.

### Model export

**Fig. 17:**
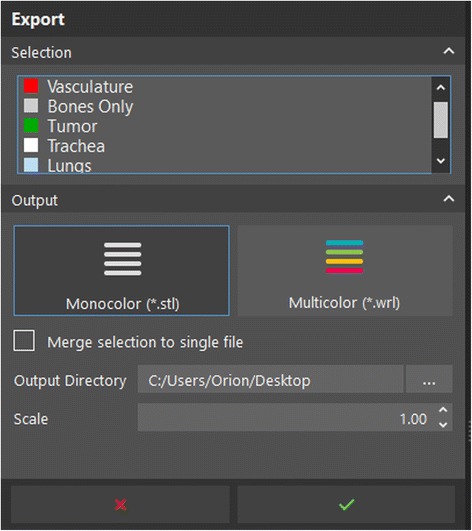
Setup of the Export operation

After model generation is complete, export the models to STL files using the ‘Export’ tool from the ‘Prepare Print’ menu (Fig. 17). Add all the parts from the Parts list by left-clicking them, select the Monocolor (.STL) output, and choose the appropriate export directory using the ‘…’ button. You may scale the exported models if you so wish by using a scaling factor, as demonstrated. The scaling factor requires verification every time the model is exported, as inappropriately set scaling factor may result in wasted prints.

## Creating printable models

**Fig. 18:**
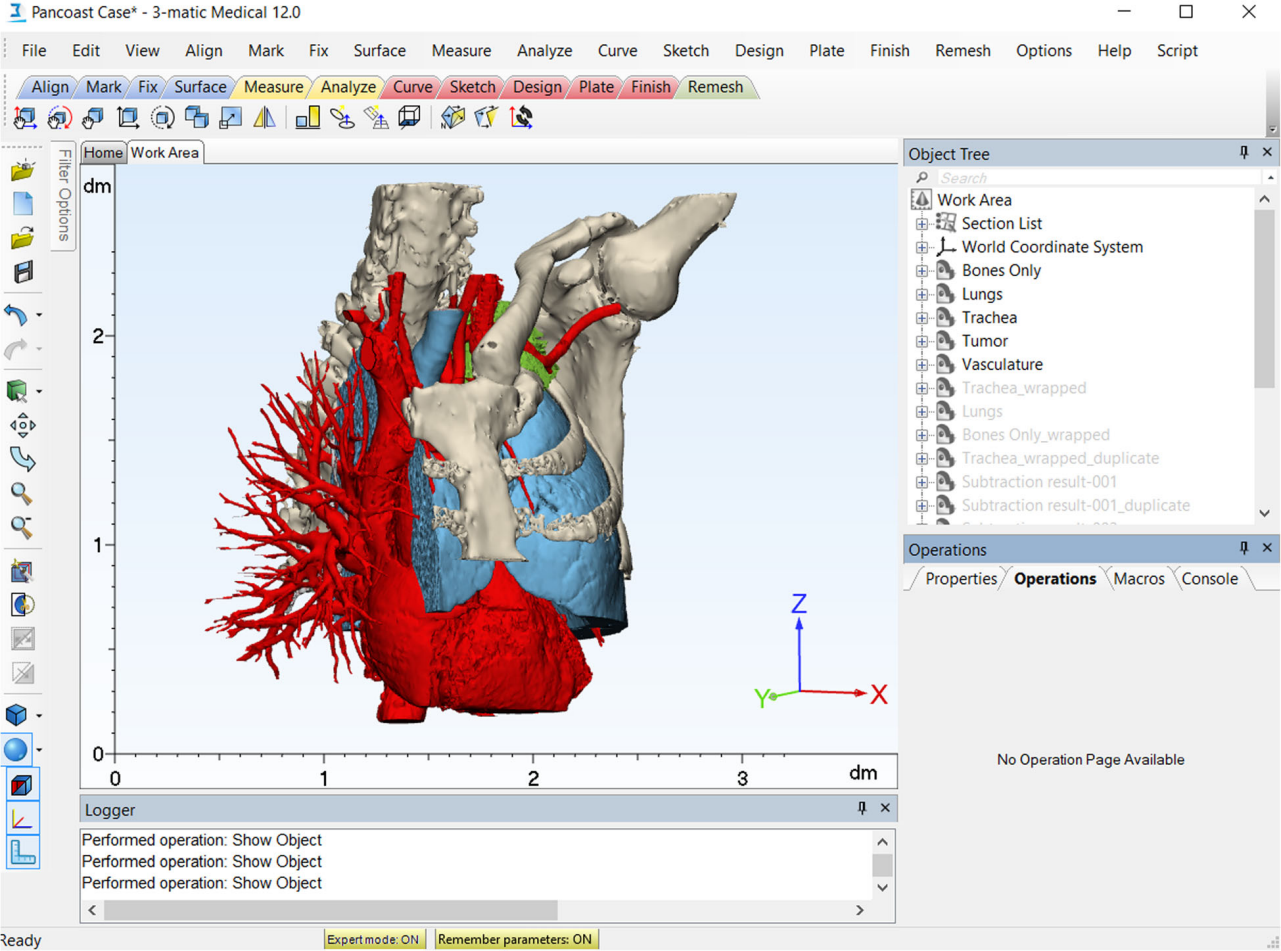
Graphical user interface of the 3-matic software. Top left, the menu and tabbed menu toolbars. Center, work area with the visualization of the current model. Right upper corner, the Object Tree with all the involved objects. Right lower corner, the Operations tab, with all operation parameters. Bottom left, the Logger which logs the current actions. Note the Expert Mode at the bottom of the window. This expert mode allows control of additional parameters, some of which are used in this work

In this part, we demonstrate the techniques involved in the generation of hollow and solid printable models using the 3-Matic CAD software. To begin, review the anatomy of the software (Fig. 18).

The basic 3D object manipulation shortcuts and controls are similar to those used in the inPrint 3D view. A set of STL files is included to carry out 3-matic manipulations, without having to create these models through the steps above. Import these STL files using the File > Import Part… menu to begin. The resultant models will appear in the Work Area. Model colors may vary, but may be adjusted through the Properties tab, Front Face color setting.

### Creating hollow airway models

You will notice that while the trachea and vessels are hollow structures, they are represented as solid, corresponding to the air and contrast filling them, respectively. To rectify this, we will use the Wrap tool to smooth and correct these models, followed by the Hollow tool on each of these models to simulate the tracheal and vessel walls, respectively. While the Wrap tool corrects and applies smoothing to the model to create a watertight, printable object, the Hollow tool creates hollow objects based on solid objects by creating a wall on the outside, inside, or both aspects of the outer surface of the solid object. In this case, we will create a 3 mm-thick outside wall for each of these structures. Selection of the wall thickness may be either arbitrary, aesthetic, or guided by material and practical considerations, and in this case is arbitrary, for demonstration purposes.

**Fig. 19:**
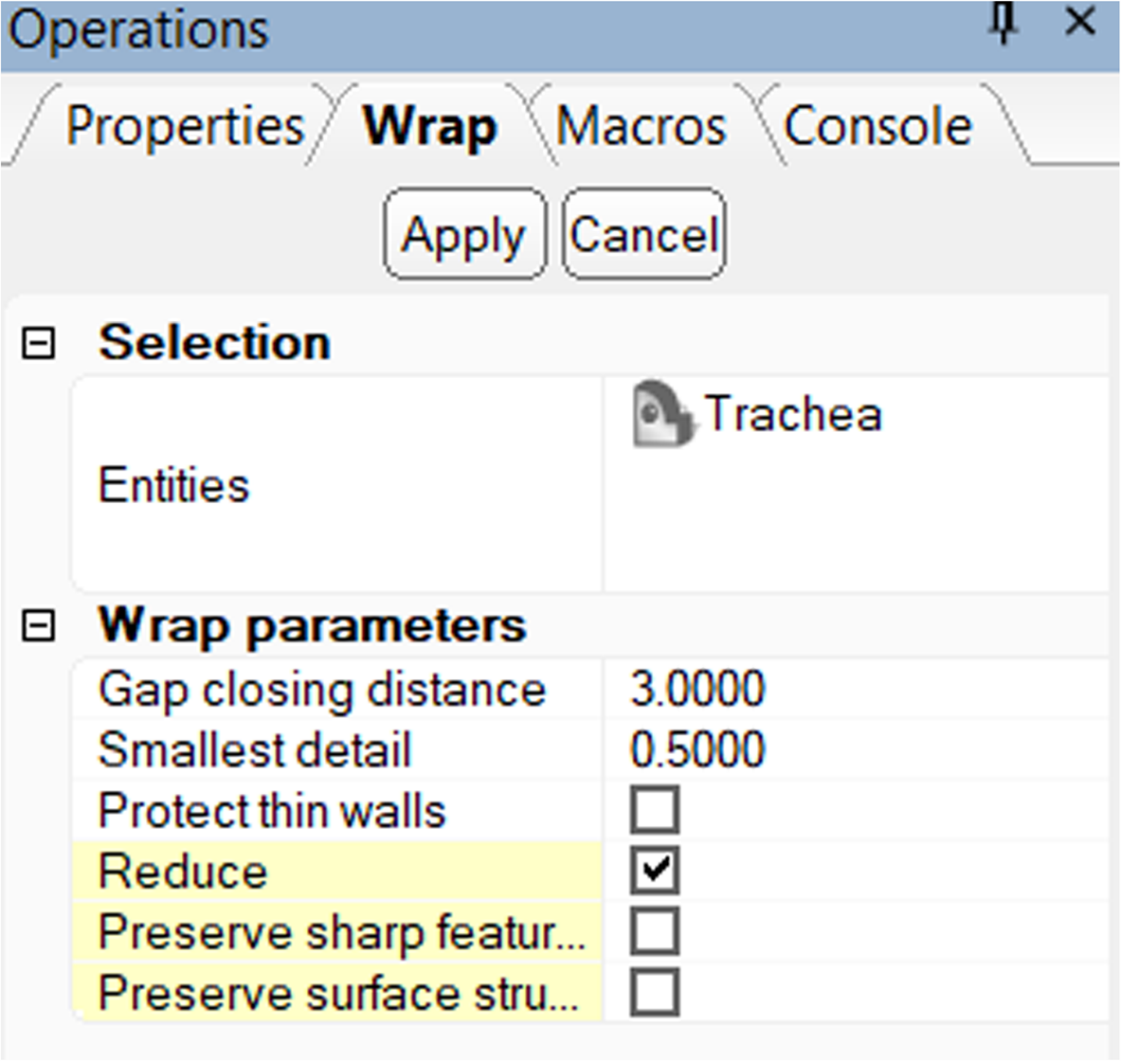
Setting up the Wrap operation for the trachea.

To do this, right-click every object that is currently not needed in the Object Tree and in the menu that appears, select the ‘Hide’ option. This will clear the scene and help visualize the edited model better. Starting with the trachea, left-click this object in the Object Tree and then select the ‘Wrap’ operation from the ‘Fix’ menu. In the Operations tab, set the gap closing distance to 3 mm and the smallest detail to 0.5 mm (Fig. 19).

**Fig. 20:**
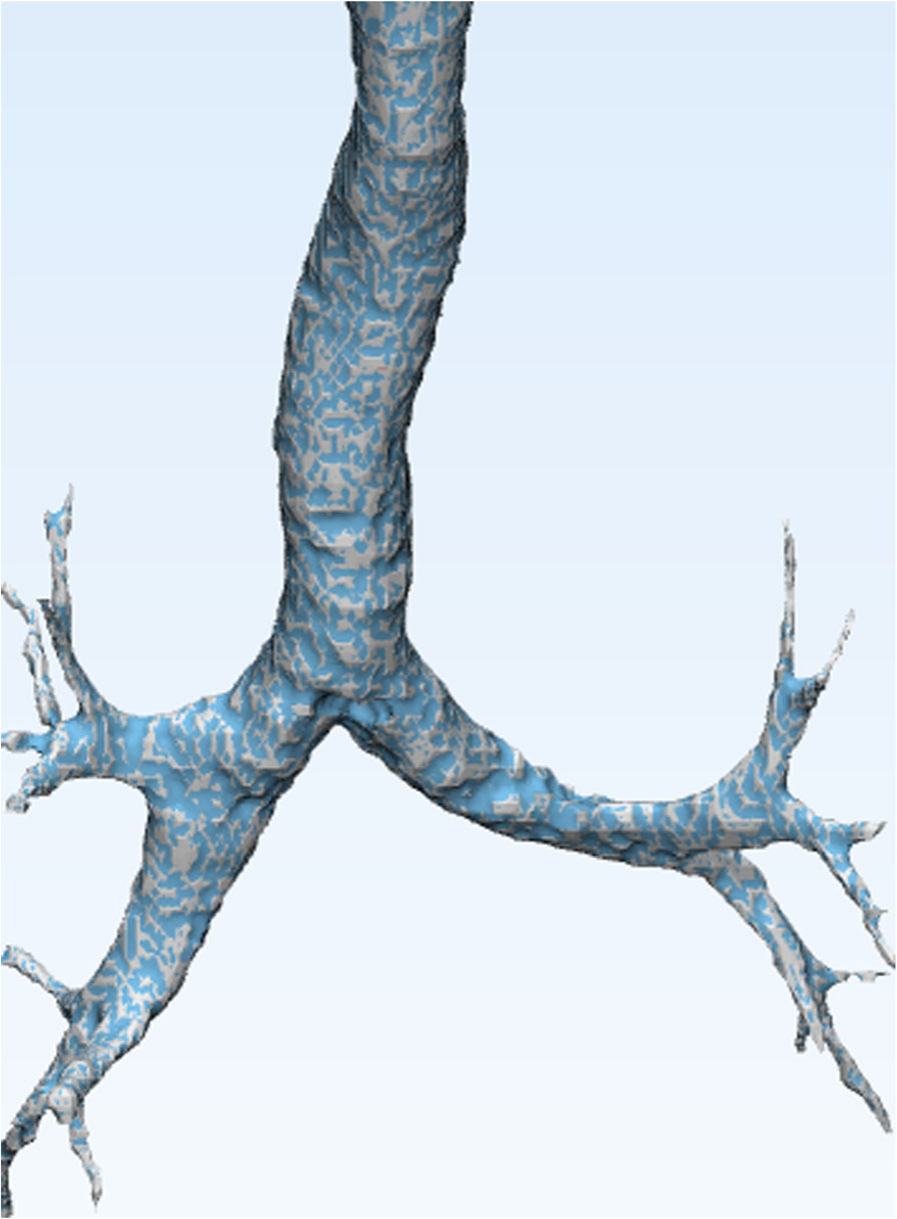
The smoothed and wrapped tracheal model (blue) overlapped with the original model (grey)

This will eliminate all gaps within this structure introduced by artifact such as beam hardening and high image noise. Moving forward, when the operation parameters are set up, use the ‘Apply’ button to execute it. The resultant model, titled ‘Trachea_wrapped’ appears on the Object Tree. You will notice a significant overlap with the existing model, with many of the imperfections of the previously created model smoothed as part of this operation (Fig. 20). Note that it is possible to create a model that is too highly wrapped or smoothed to reflect anatomy adequately, and verification of the final model in the context of the original DICOM images is required to ensure accuracy. Hide the original trachea model (right click it on the Object Tree and select ‘Hide’).

**Fig. 21:**
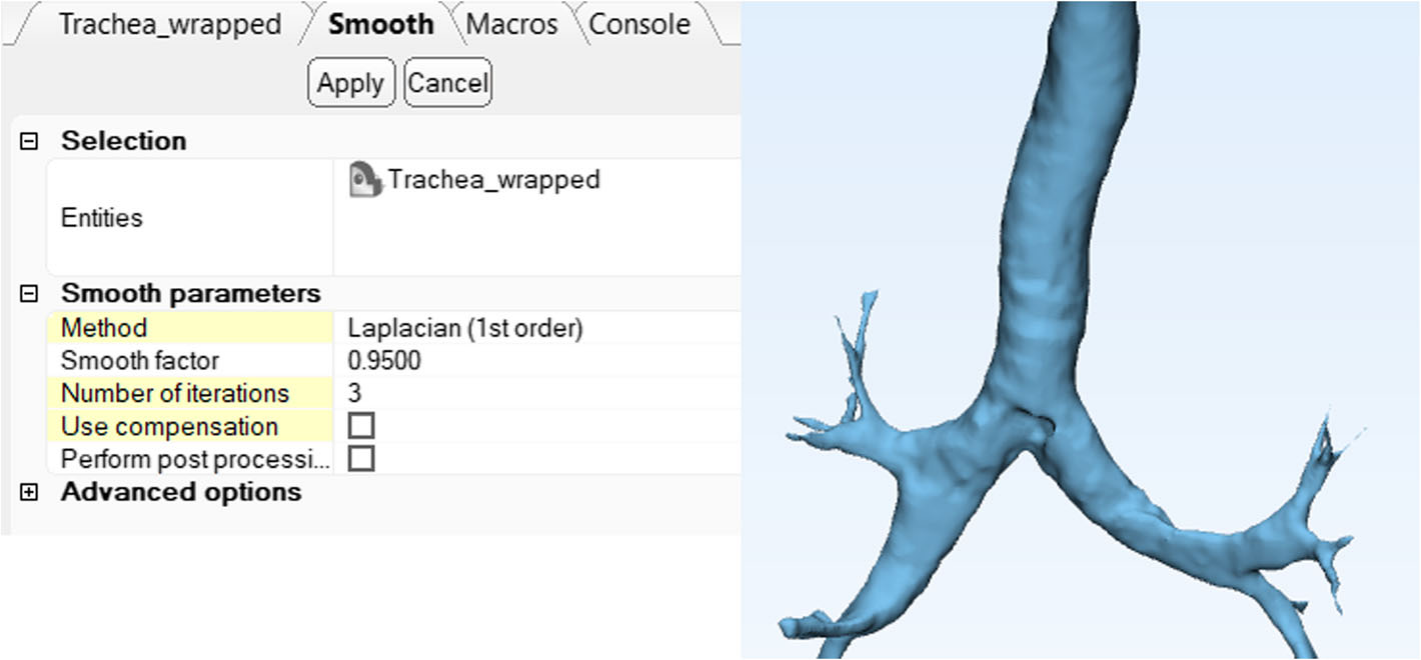
Setup of the smoothing operation. Note the yellow settings, which are the advanced settings that can be turned on or off using the Expert Mode toggle at the bottom of the window

Now, use the smoothing tool through Fix > Smooth in order to apply smoothing to the trachea using the parameters specified, including the advanced parameters highlighted in yellow (Fig. 21).

**Fig. 22:**
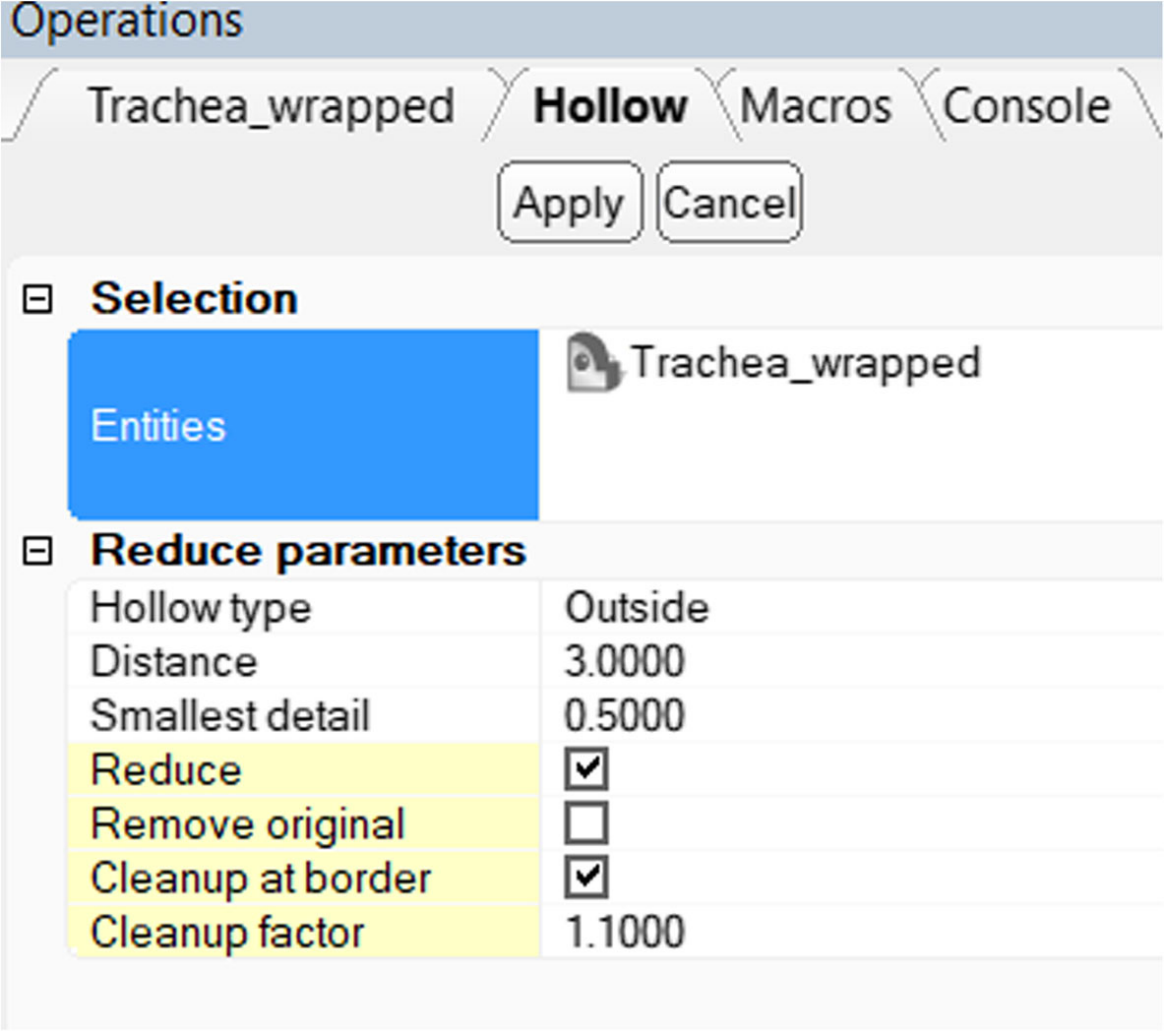
Setting up the hollow operation

Since the trachea is a hollow structure with its wall on the *outside* of the intraluminal air segmented earlier, we will use the ‘Hollow’ tool from the Design > Hollow menu as specified to generate a hollow structure with a 3 mm outside wall (Fig. 22).

**Fig. 23:**
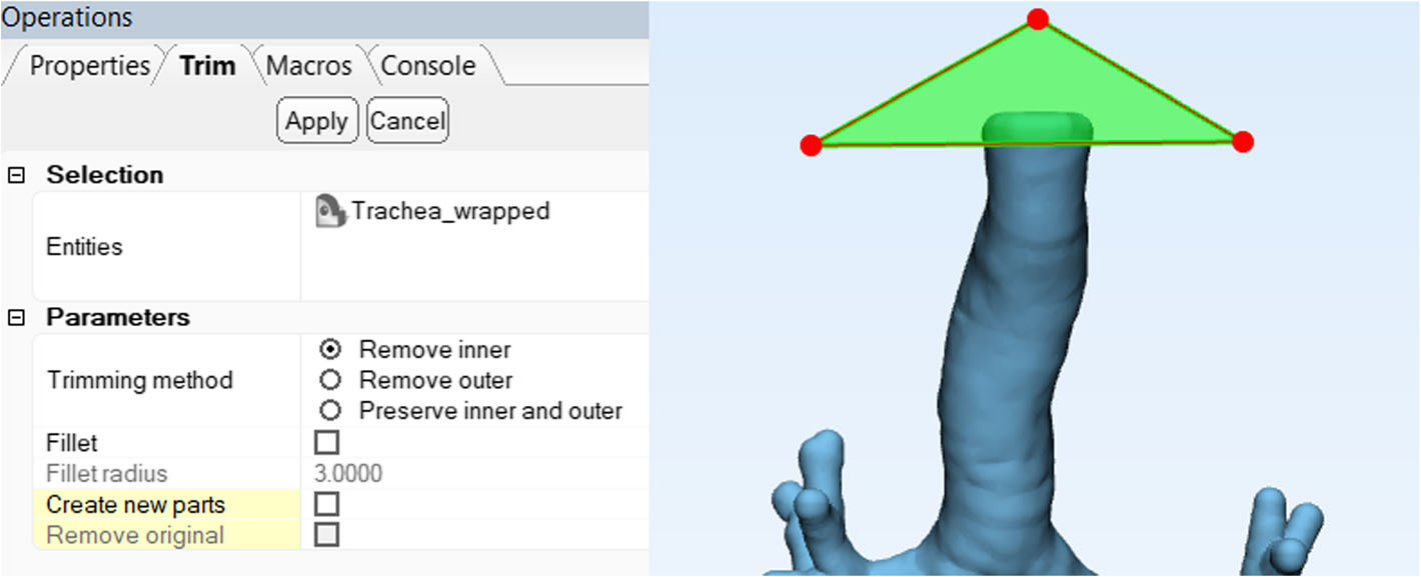
Setting up the Trim operation

You will notice that the Hollow operation has resulted in a hollow structure with no external opening. In order to simulate the airway, we will cut off the top of this hollow structure using the Finish > Trim tool. First, position the trachea on the frontal view. To do this, right-click anywhere in the Work Area and select View > Front. Then, using the Finish > Trim tool, ensure that the operation is set up as indicated and the Trachea_wrapped model is in the Entities List. Draw a triangle or any other shape that transects the upper portion of the trachea, as demonstrated by left-clicking at the indicated locations and closing the shape at the end by clicking the starting point again a single time (Fig. 23).

**Fig. 24:**
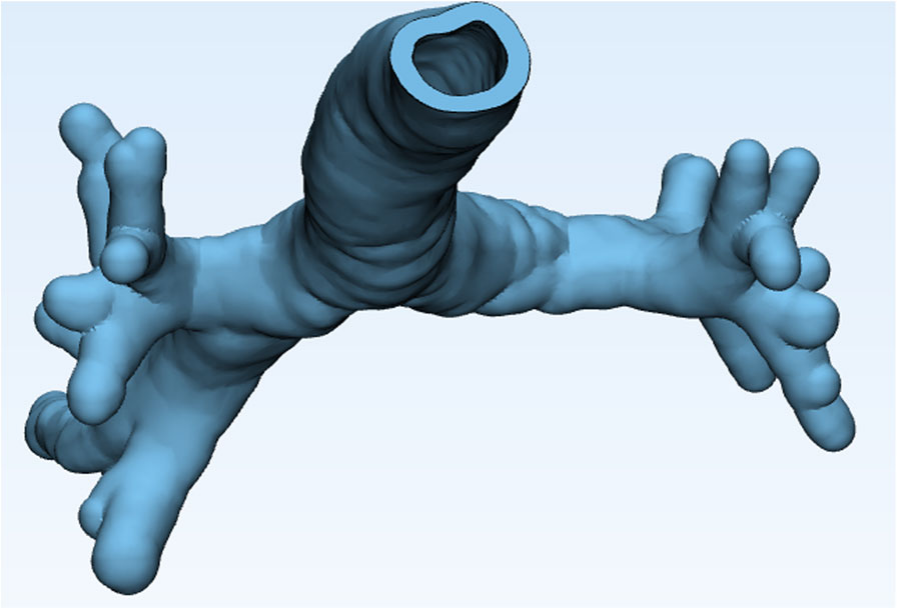
Hollow model of the trachea.

The result of this operation is a hollow model of the trachea (Fig. 24). The same operations can be reproduced to create hollow models of any structure, including the vasculature and the alimentary tract, among others.

### Preparing solid bone and lung models

**Fig. 25:**
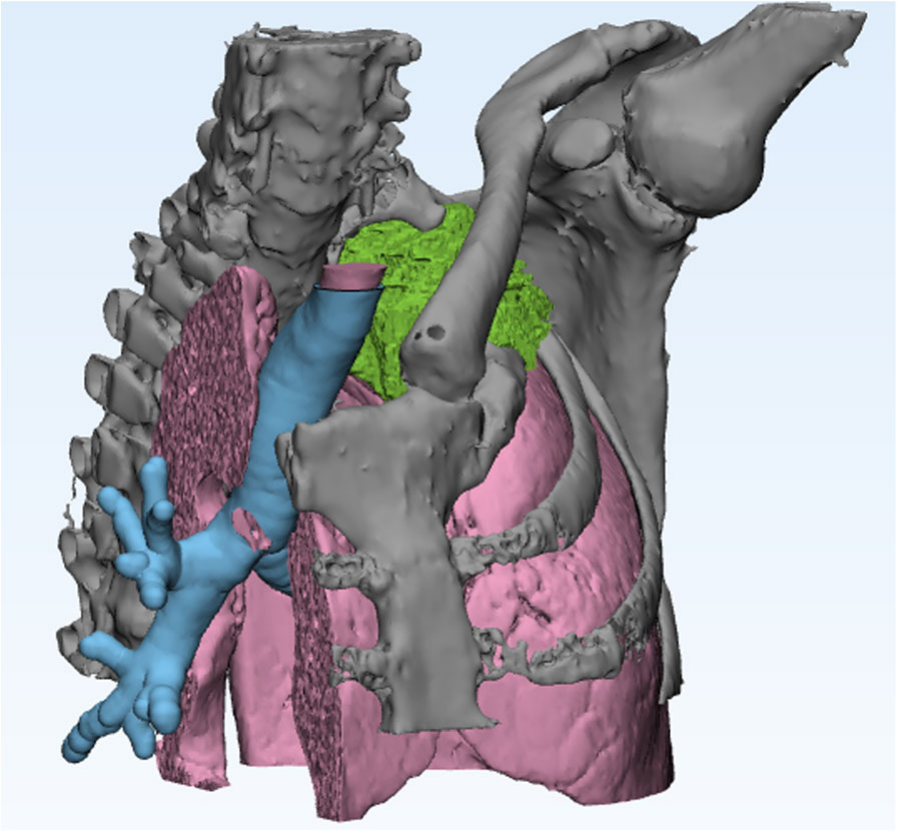
Sample output of the wrapping and smoothing operations on the model elements. Individual results may vary based on the selected parameters

The smoothing and wrapping tools can be applied to the remaining models, using the Wrapping tool with a lower gap closing distance of 1.5 mm (chosen to balance computational time and quality), followed by the Smoothing tool as indicated previously. The result is a significantly smoother geometry that is easier to 3D print and post-process, but that still maintains the necessary anatomical accuracy to provide informed preoperative planning decisions (Fig. 25).

### Preparing models for printing

**Fig. 26:**
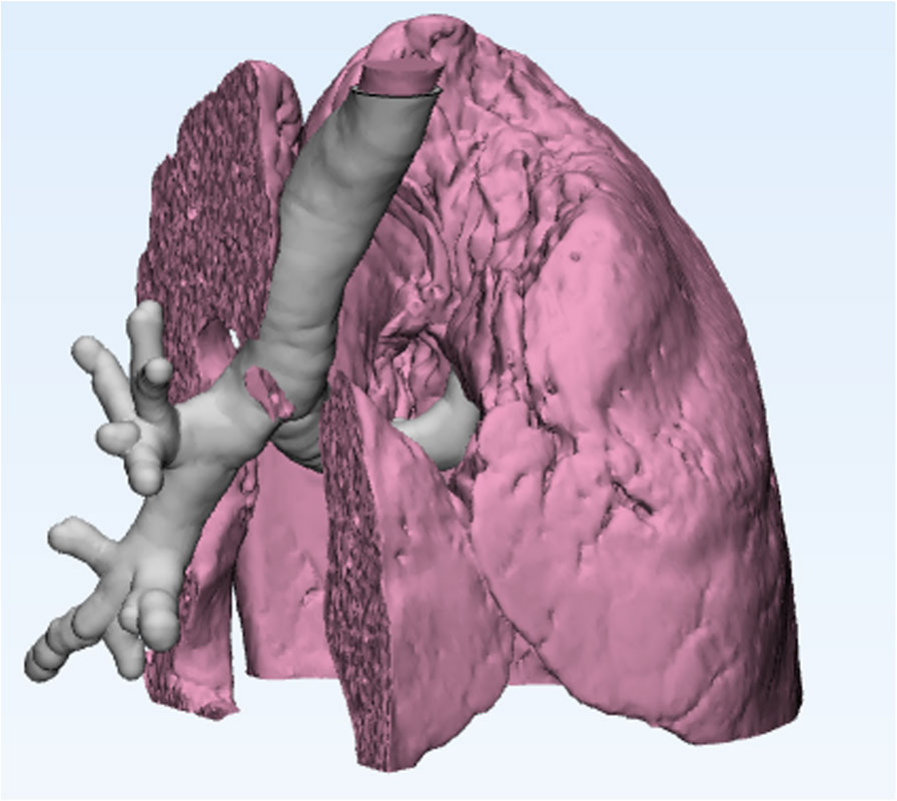
Models of the trachea and the lung. Note the significant overlap

In order to ensure model printability on multi-material printers as demonstrated here, one needs to ensure that no model parts overlap and that all extraneous loose, non-contiguous model fragments are removed. This involves either using Boolean subtraction to subtract common portions of the various model parts, or alternatively, Boolean union to unify all constituent parts into a single whole to be 3D printed as a single unit. To illustrate the process of model cleanup for printing, consider the newly produced hollow trachea and the Lungs model which also contains the tracheal lumen and a portion of the contralateral lung. With this overlap of structures, the model may not be printed correctly (depending on the printer used), and inclusion of unneeded portions is wasteful (Fig. 26).

**Fig. 27:**
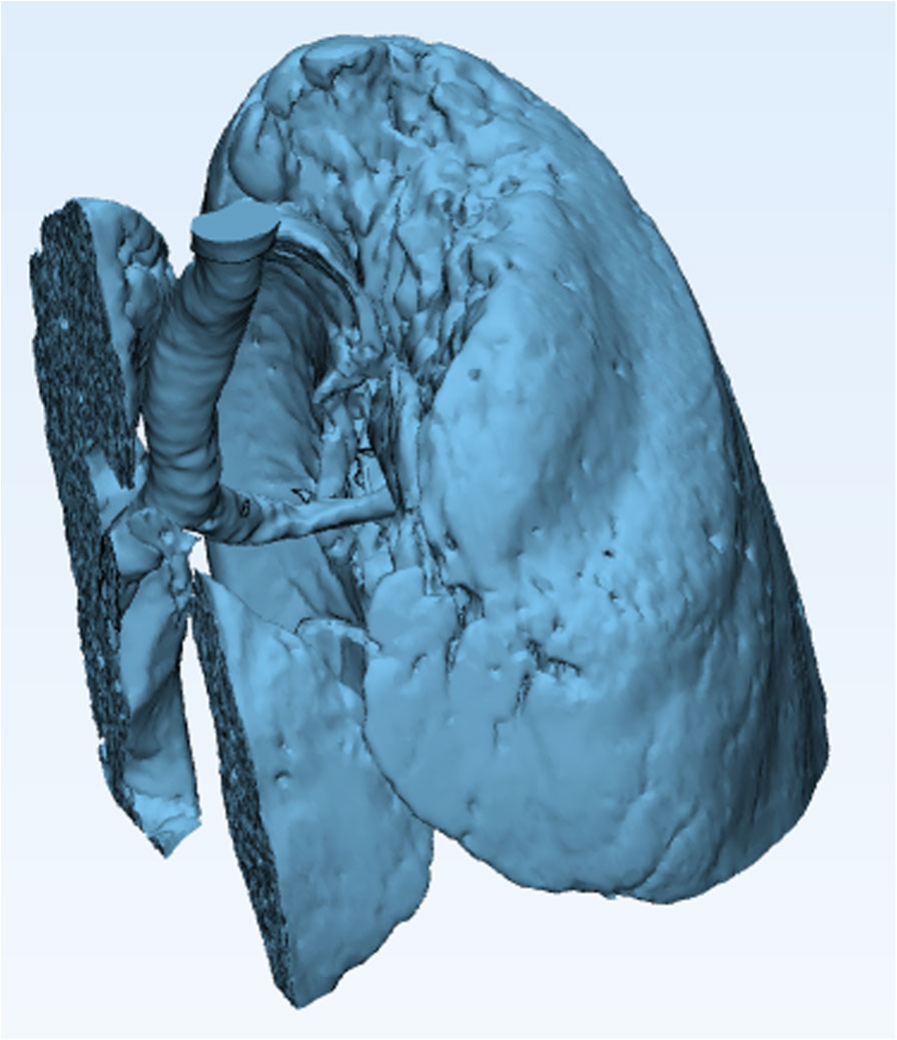
Lung model following subtraction of the hollow trachea.

To rectify this, first visualize the Trachea and the Lungs models by right clicking them and clicking ‘Show’ from the Object Tree. Next, use the Design > Boolean Subtraction tool. This tool allows subtraction of one model from another. For the ‘Entity’, or the model to subtract from, choose the Lungs model, while the ‘Subtraction Entity’ will be the Trachea_wrapped model, resulting in a model as shown (Fig. 27).

**Fig. 28:**
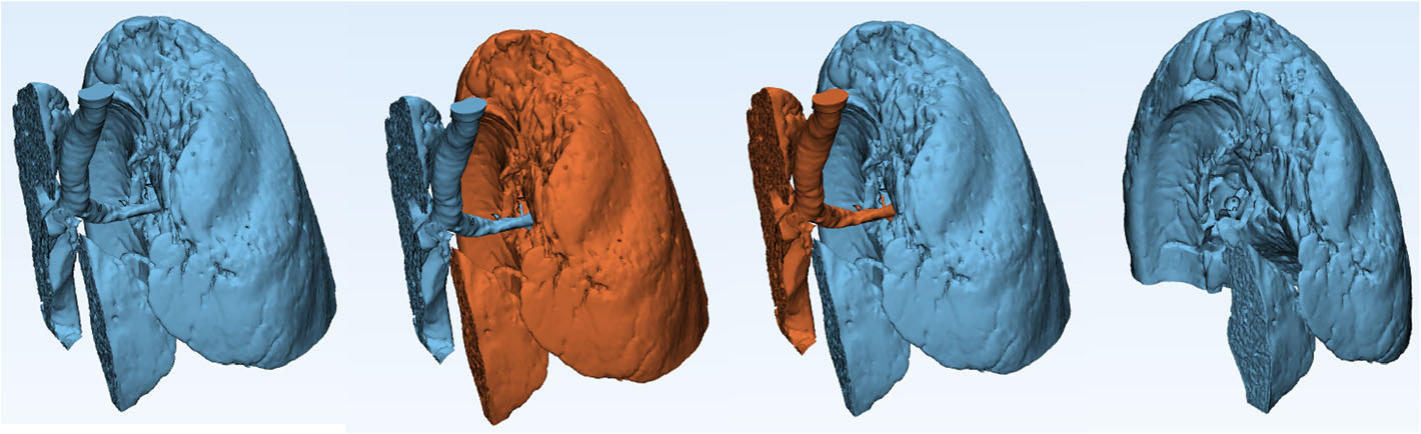
Selecting the non-contiguous portions of the lung models and deleting them

Note that this model contains a number of disconnected fragments. Use the Mark > Shell tool to mark the left lung that we wish to keep. Use the Mark > Invert tool to select all the other parts, and press ‘Delete’ on the keyboard to remove these (Fig. 28). The same process can be repeated with all model parts to ensure no overlap.

In a clinical production environment, individual models may be labeled using the Finish > Quick Label tool, and further inspected for quality issues using the Fix > Fix Wizard tool. The final STL models may also be simply copied by selecting them from the Object Tree using the CTRL + C shortcut and pasted into the inPrint or Mimics software with the original DICOM images using the CTRL + V shortcut for further inspection of their accuracy.

## Computer-aided design

While model post-processing is quite useful in optimizing models for printing, the role of Computer-aided design (CAD) is better exemplified by demonstrating an approach for the creation of a customized tracheal stent for a simulated case of significant tracheal stenosis secondary to mass effect from an adjacent mediastinal tumor (included in Additional files 1, 2 and 3).

### Generating a customized tracheal stent

**Fig. 29:**
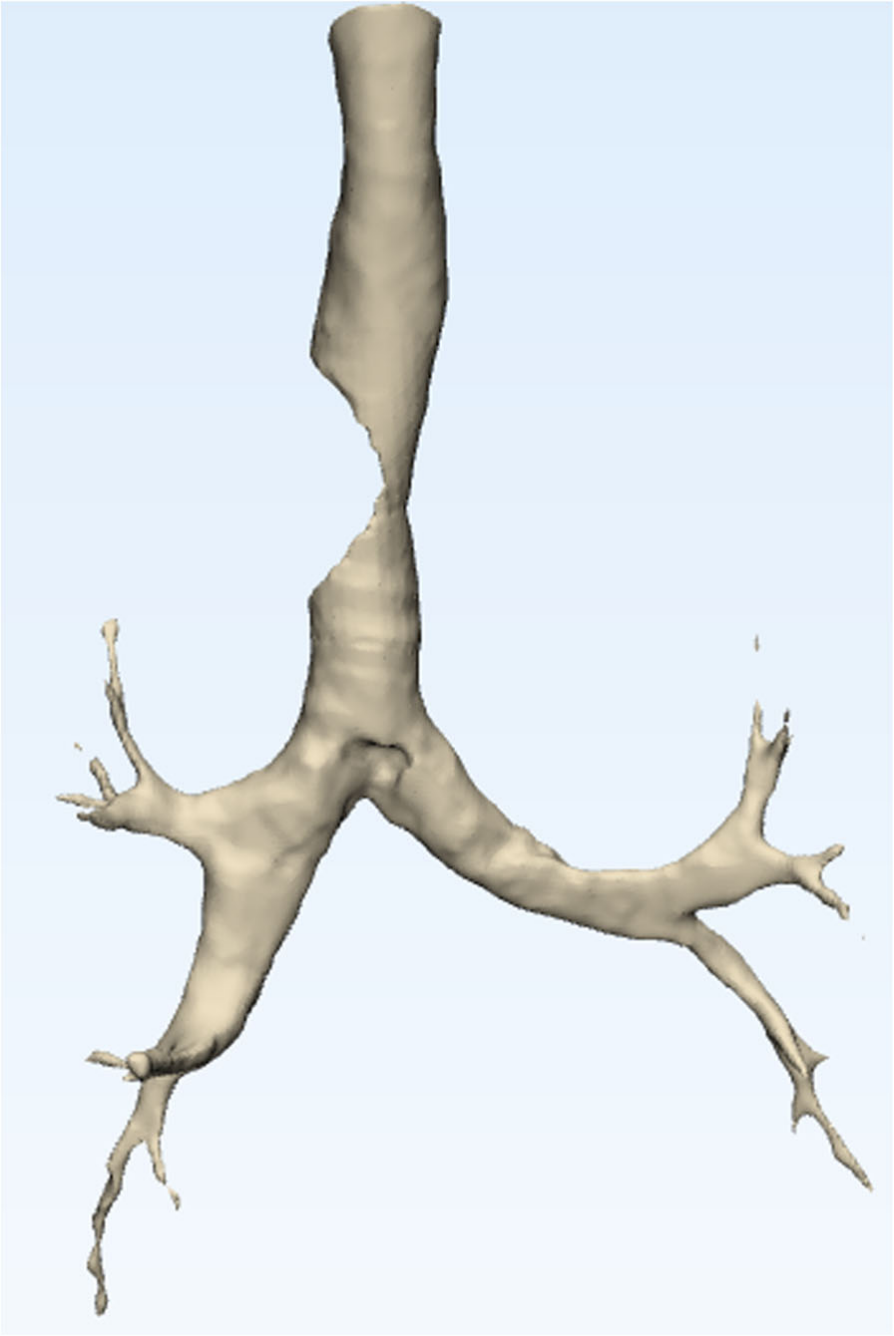
Tracheal model with simulated mass effect

We will start from a model of a patient’s airway segmented from CT images to include air density HU range, followed by solid model creation. This model is available as additional data enclosed with this work. We shall begin by importing this solid airway model using methodology outlined in Section 2. The mass effect on the airway is from a simulated adjacent tumor, not shown (Fig. 29).

**Fig. 30:**
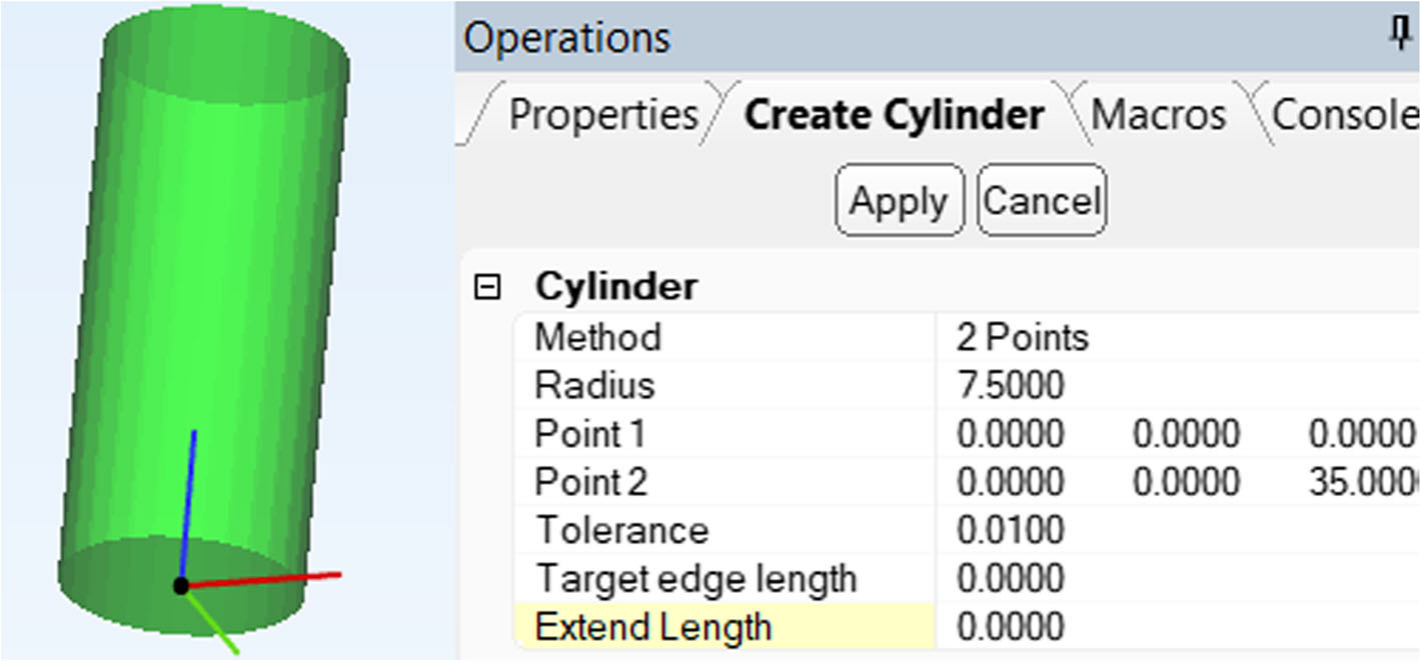
Create Cylinder operation. Note, you may need to find the origin within the Work Area to see the new cylinder

We use a cylinder primitive to fill in the cavity left behind by the tumor. From the Design > Create Primitive menu, select the Create Cylinder option and set up the parameters as demonstrated, with a cylinder 35 mm long and 7.5 mm wide (Fig. 30). These values were approximated by measuring the model directly using the tools in the Measure menu.

**Fig. 31:**
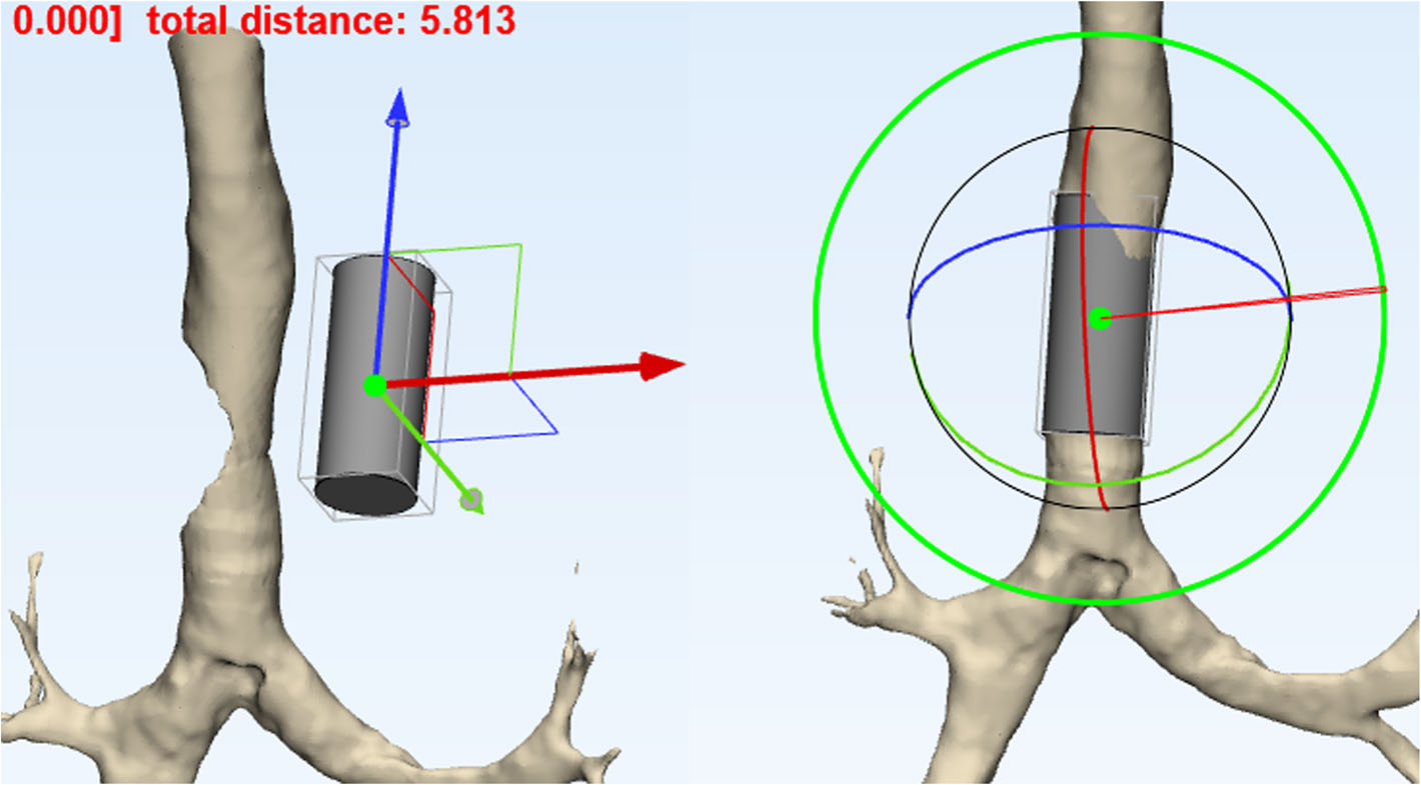
Interactive rotation and translation, achieved by dragging corresponding arcs or axes in the model-associated orthogonal planes

This cylinder will be positioned into place using a combination of the Align > Interactive Translate and Align > Interactive Rotate functions, until the cylinder precisely reconstitutes the luminal defect (Fig. 31).

**Fig. 32:**
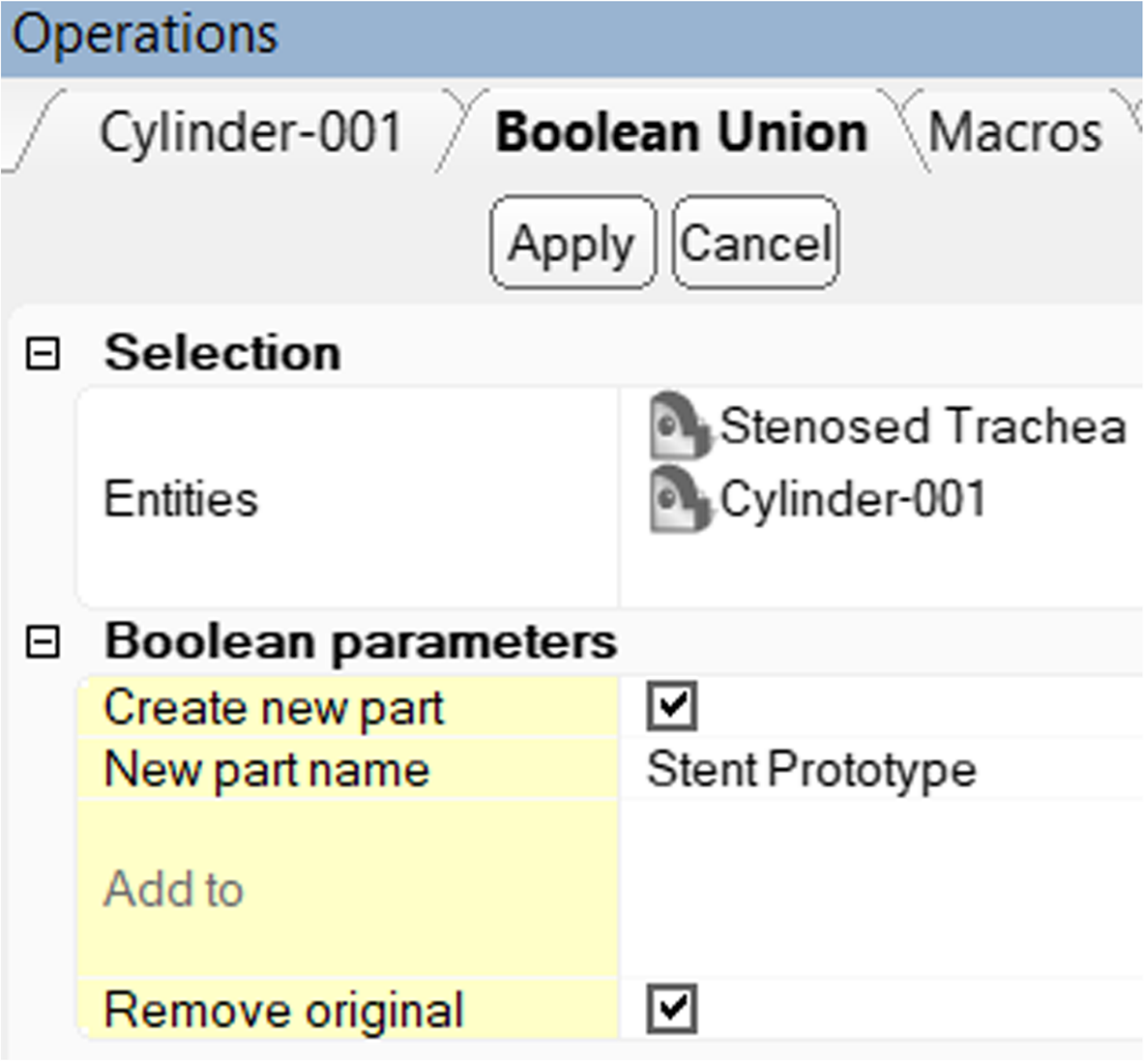
Boolean Union operation to create the first approximation of the stent prototype

The resultant model, consisting of a cylinder and the original airway can then be fused into a single entity, renamed ‘Stent Prototype’, using the Boolean Union function to generate a model of an intact airway (Fig. 32).

**Fig. 33:**
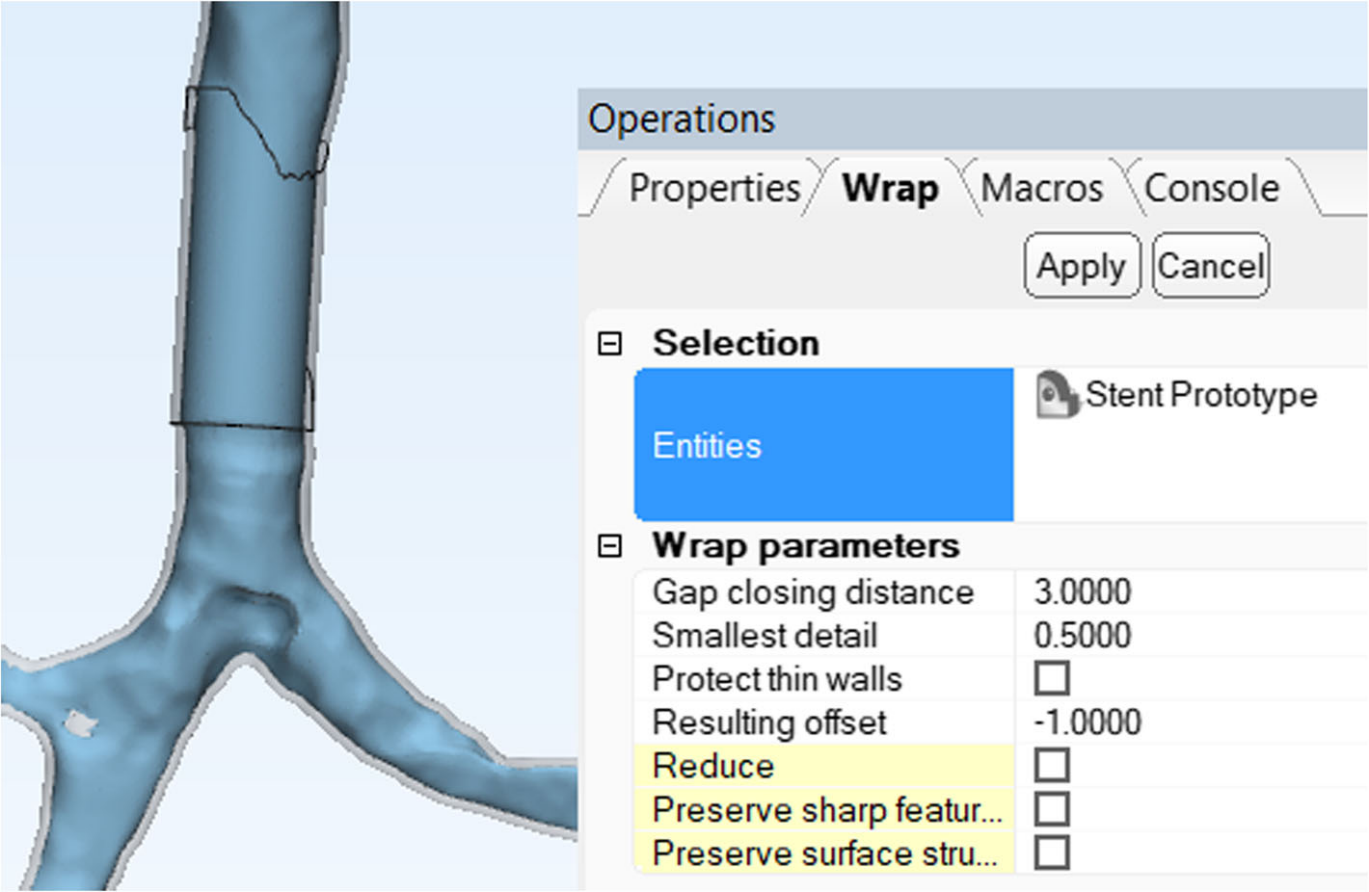
Setup and the result of the Wrap operation (blue) with a -1 mm offset in relation to the original Boolean Union result (grey, transparent)

To ensure the intended stent can be placed within the trachea, it needs to conform to the inner lumen of the trachea, but not exceed it. Thus, a degree of scaling or offset is required. While this is possible with a dedicated Scaling function in 3-matic, this can be achieved during object wrapping with an offset parameter specified as -1 mm (Fig. 33). This effectively ensures a 1 mm inner offset for the newly created, wrapped part and acts as an equivalent of scaling the model to a size that is amenable for placement within the airway. Smooth this part as previously indicated.

**Fig. 34:**
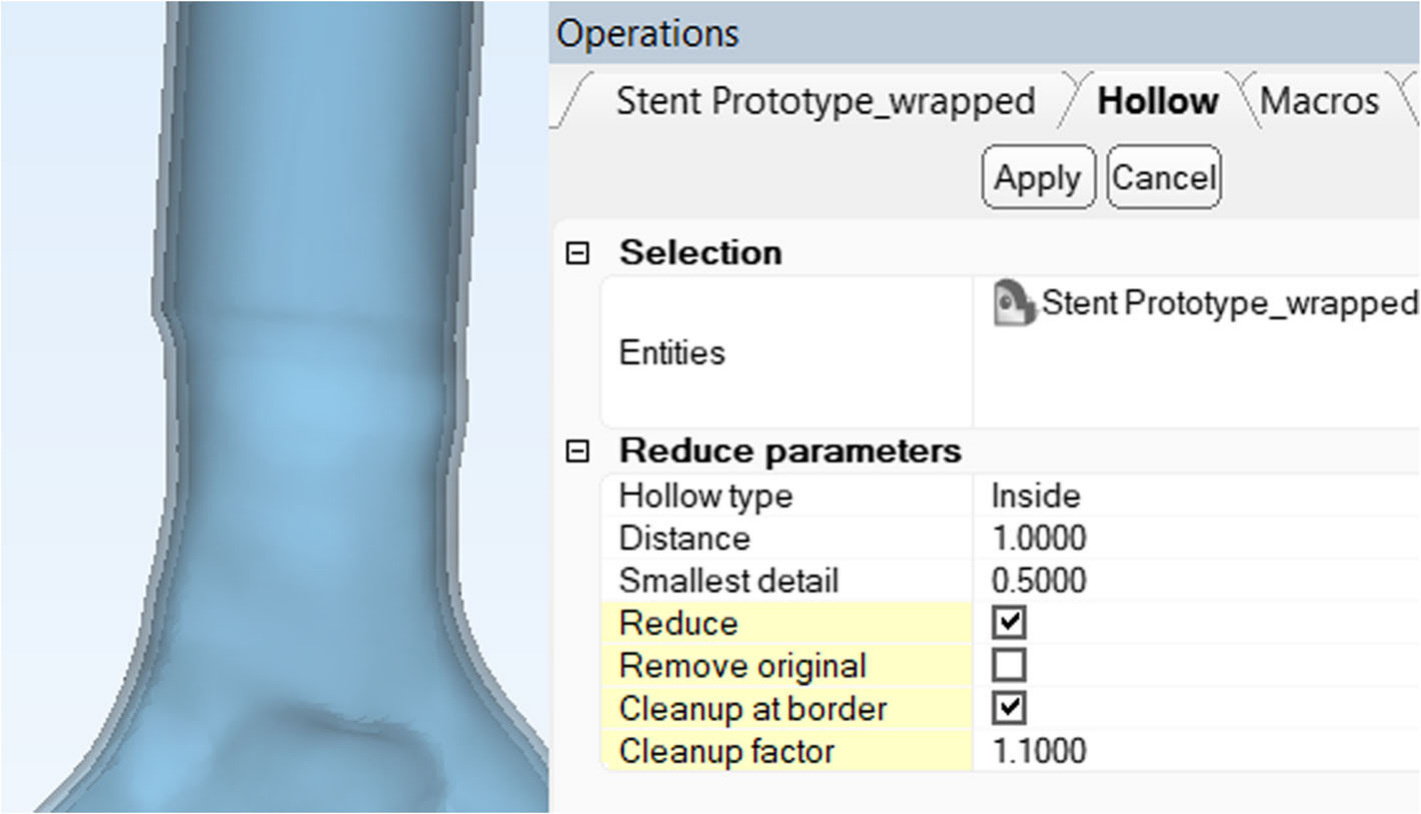
Setup and result of the Hollow operation

The resultant scaled, solid model with smoothed transition between the cylinder and patient’s trachea, is ready to simulate a stent. Since the stent is a hollow structure, we will use the methodology of Section 2.1 to create this hollow structure, this time opting to create 1 mm walls on the inside of this model (Fig. 34).

**Fig. 35:**
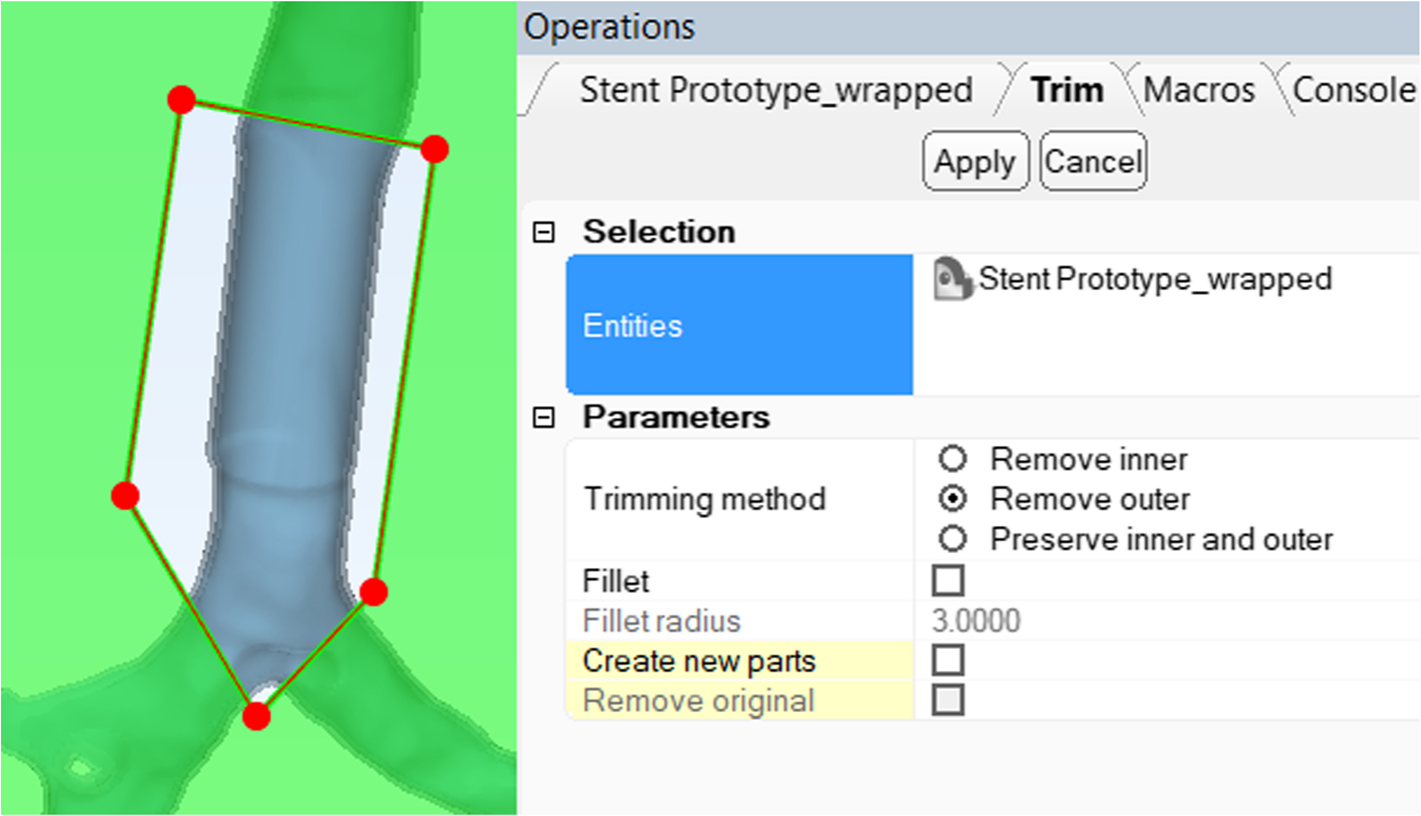
Setting up the trimming operation to create a hollow stent

**Fig. 36:**
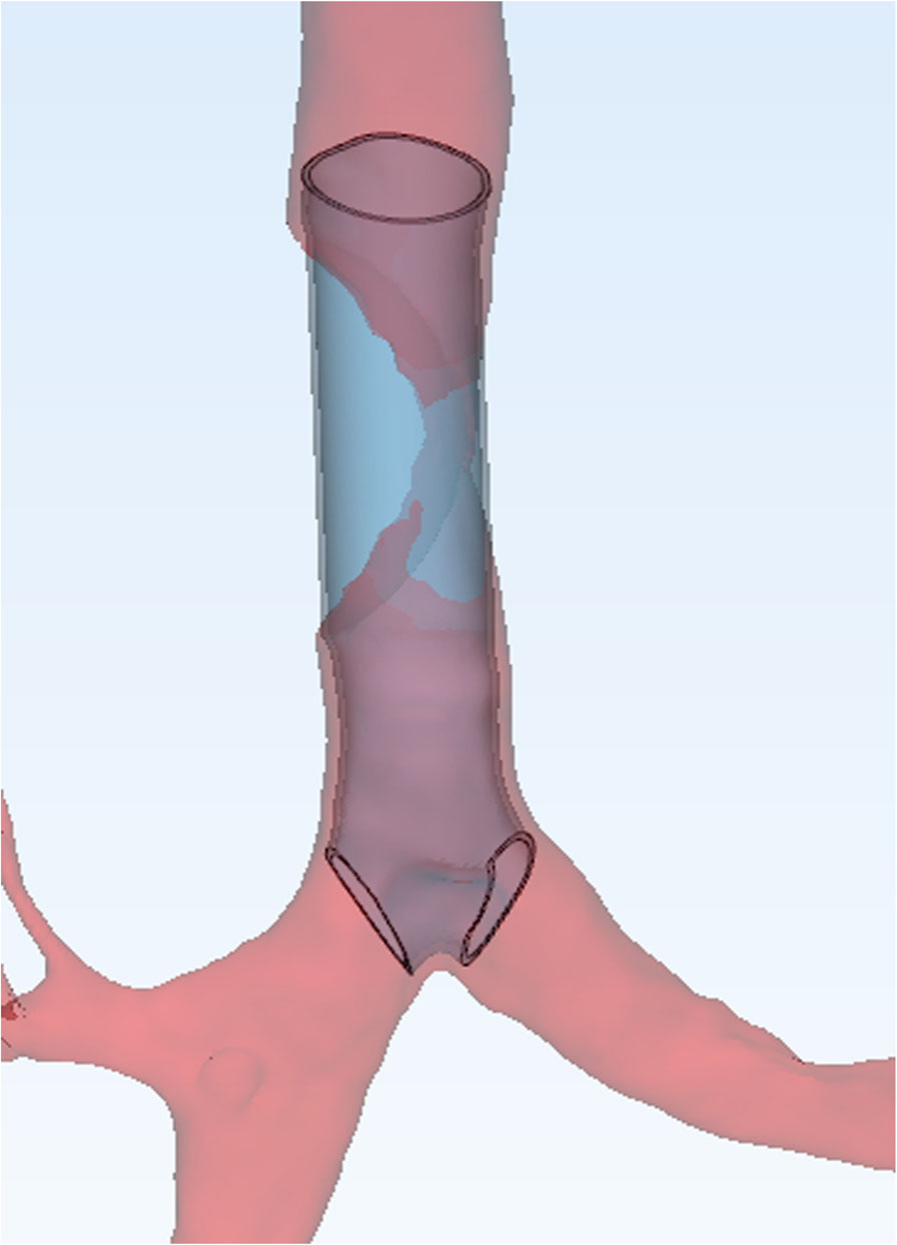
Final stent (blue) inside the original airway model (red), demonstrating adherence to the lumen

Trim the stent to desired size using the Finish > Trim function as indicated, opting to remove all parts outside of the shape demonstrated (Fig. 35), making a ready-to-print model (Fig. 36).

Please note that while the approaches demonstrated here are highly simplified and would not be used without further refinements, they illustrate principles of CAD. Alternative approaches to achieve similar results exist, and may, for example, involve drawing the desired configuration of the trachea and main stem bronchi directly on the DICOM images during the segmentation step using the Brush tool in inPrint, as discussed earlier, or using midline symmetry to replicate the native configuration of the trachea as discussed in the 2016 course [2].

### Advanced CAD: Injection molding design

Combinations of the tools discussed above can be applied to create complex patient-specific guides using primitive geometrical shapes as well as 3D models obtained by scanning medical devices and surgical guides, or importing their premade CAD models. With the ability to deform and align such objects as necessary, the range of possible applications of the CAD tools discussed is quite wide.

As an example, consider the creation of injection molding for the patient-specific stent designed above. Molding is highly beneficial where rapid and inexpensive model replication is desired, for example in educational settings where a large number of models are used such that model destruction is expected as part of the process. Parts and devices that are expected to undergo a high replacement rate can also be replicated using molding. Note that although the precise design considerations for mold optimization are numerous, the intent of this work is to expose the reader to a basic approach that can be subsequently optimized in practical applications.

Although there are a wide number of approaches to 3D printed model replication using resin molds, including direct creation of molds starting from the desired 3D printed model, we will demonstrate the de novo creation of a mold using CAD tools. This process will consist of the design of an outer shell and the appropriately positioned inner shell, such that the molded part is retrievable and functional without the destruction of the mold, which can then be reused for multiple additional parts with a variety of resins of different colors and material properties.

**Fig. 37:**
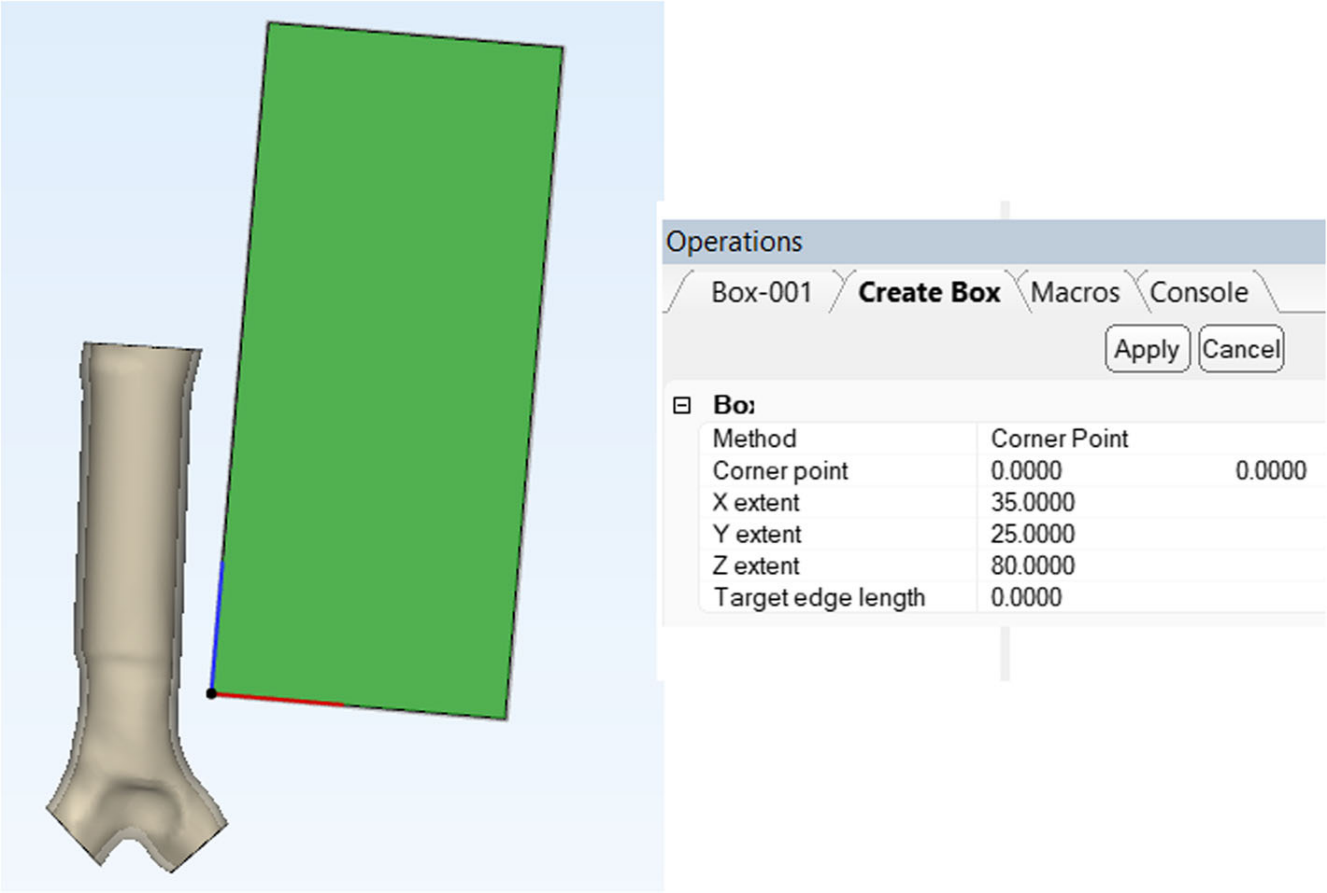
Setup for the Create Box operation

To begin, create a box primitive with parameters as demonstrated (Design > Create Primitive > Create Box), ensuring complete encapsulation of the target part with the primitive geometry (Fig. 37).

**Fig. 38:**
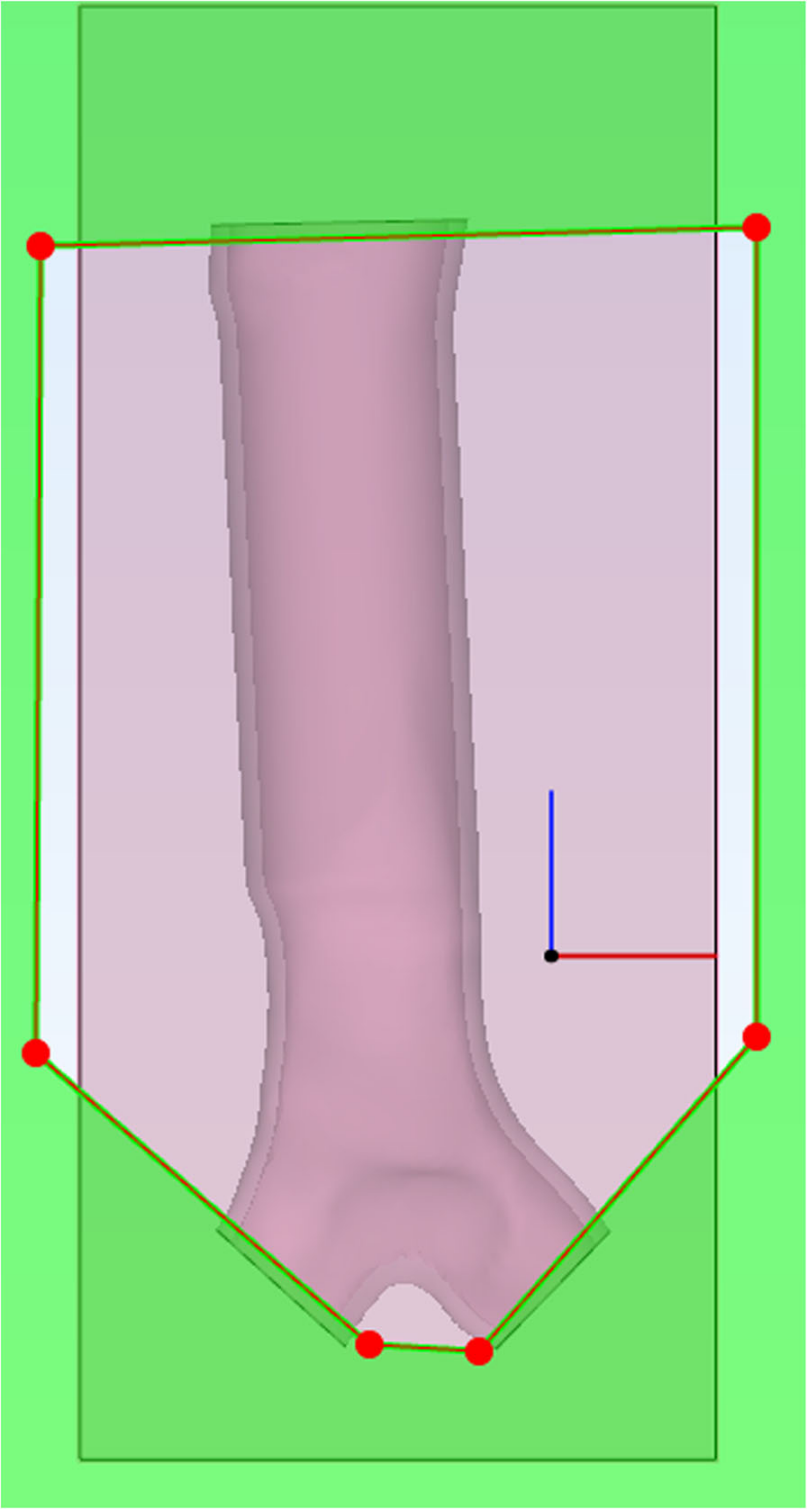
Using the trimming function on the result of subtraction of the stent from the box primitive

This primitive is then moved to overlap the stent model using the free translate tool discussed earlier, and the stent model is subtracted from the primitive using Boolean Subtraction, resulting in a hollow structure amenable for trimming along the outer edges of the stent (Fig. 38).

**Fig. 39:**
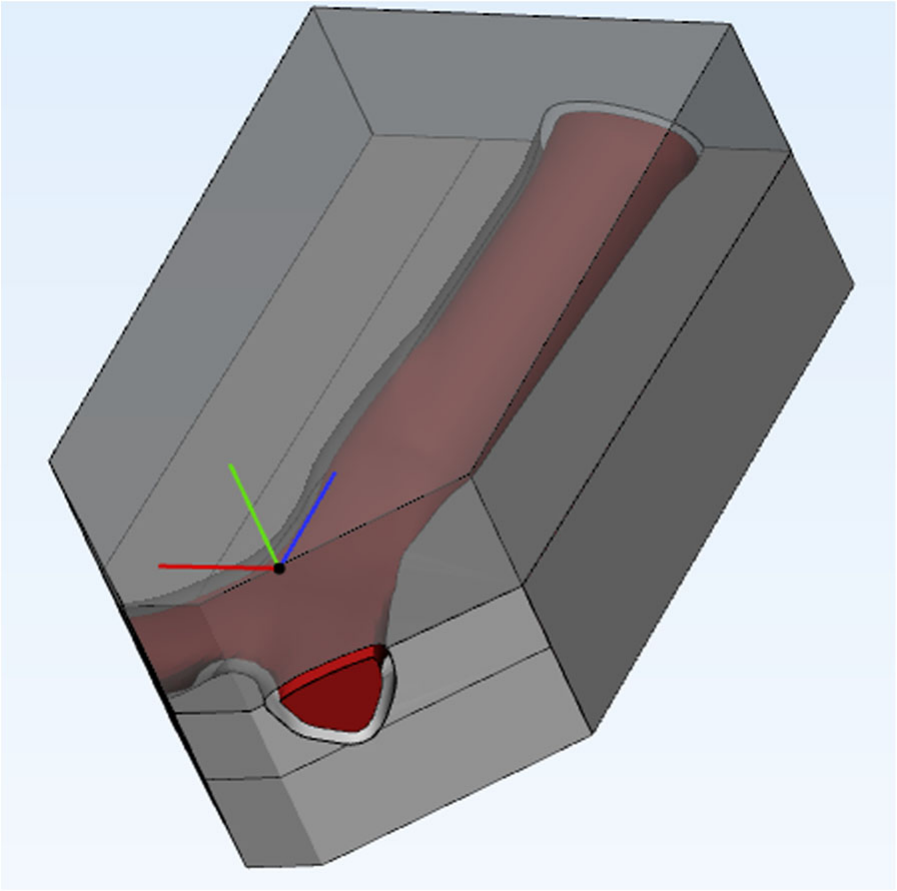
The result of separation of inner and outer portions and trimming the outer portion

The resultant trimmed Boolean Subtraction product consists of two shells – the inner shell that outlines the internal portion of the mold and the outer shell that outlines the external portion. By duplicating the resultant structure (Right-Click in the Object Tree and select Duplicate), the outer and inner portions of this model can be preserved in separate models by using the Mark > Shell tool to mark and delete (Delete key on keyboard) the inner and outer portions of the model respectively. Furthermore, to create an openable external portion, it may be cut in two pieces using the Trim function with the ‘Preserve inner and outer’ option, resulting in a three-part model (Fig. 39).

**Fig. 40:**
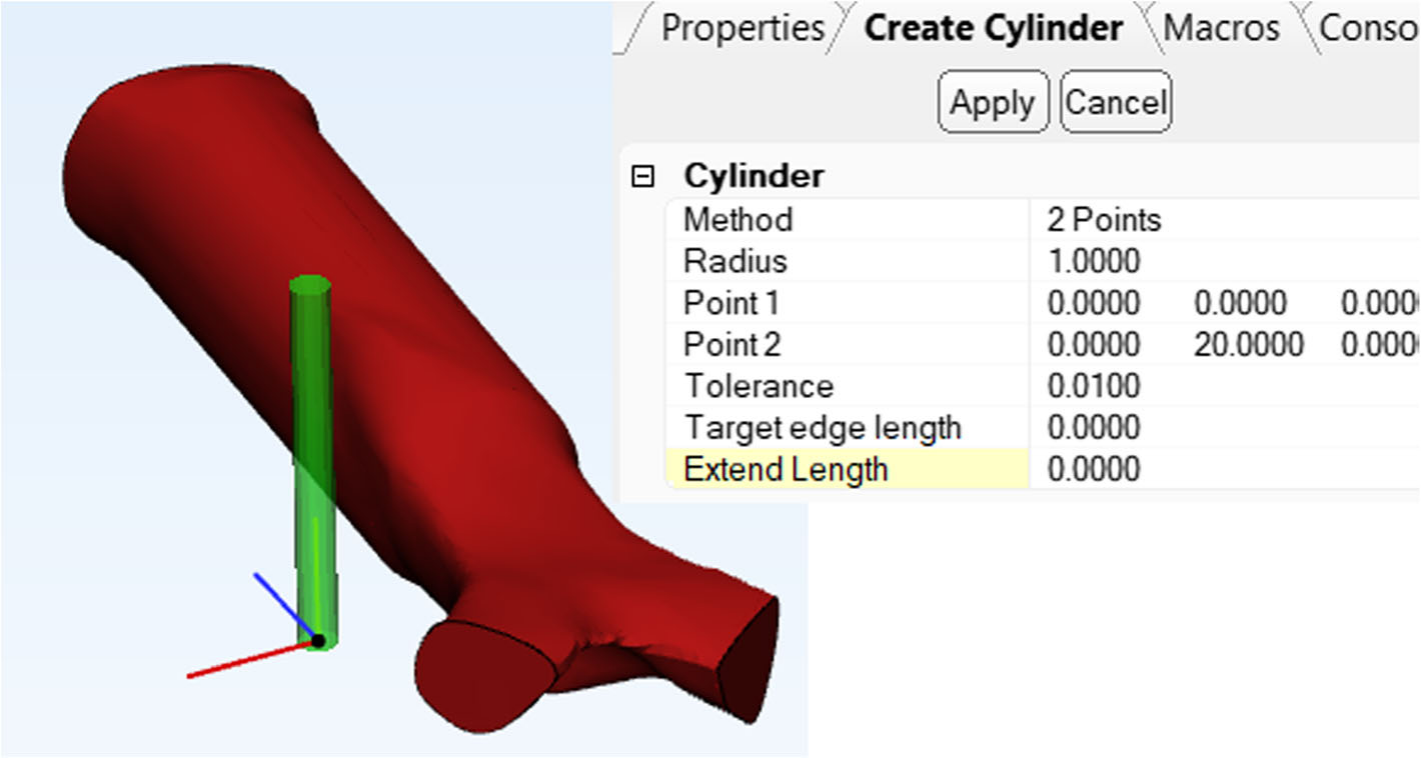
Setup for the Create Cylinder operation

To hold the inner part in place and ensure 1 mm wall thickness of the stent, support cylinders may be created using the Create Cylinder function and aligned such that the internal molding part is entirely traversed in an orthogonal axis extent (Z axis in this case), and the cylinders are within the bounds of the external portion of the mold (Fig. 40).

**Fig. 41:**
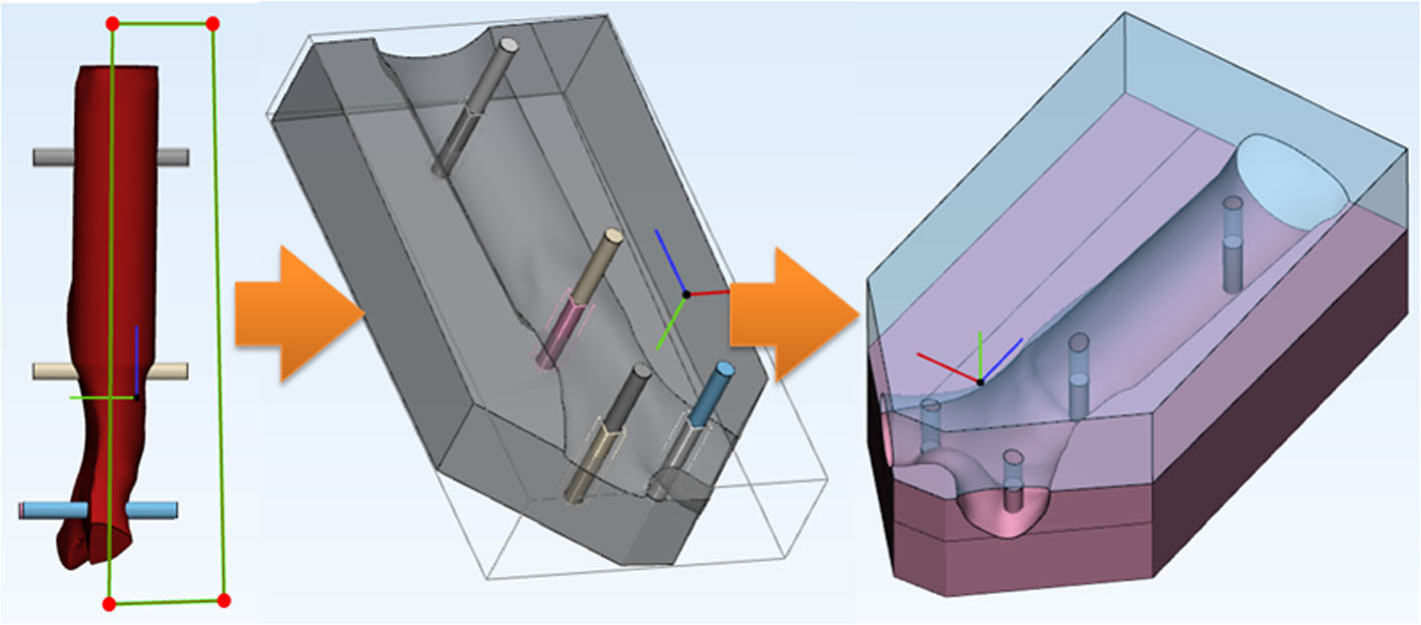
Trimming the support pins (left), using the Boolean union operation on the pins and the appropriate outer mold part (center), with the final result demonstrated (right)

In this demonstration, four cylinders were created and aligned. These cylinders are then trimmed along the midline of the internal part, keeping the inner and outer products of the trim function in place. Using Boolean Union, the appropriate supports are then united with their respective portions of the outer shell of the mold (Fig. 41).

**Fig. 42:**
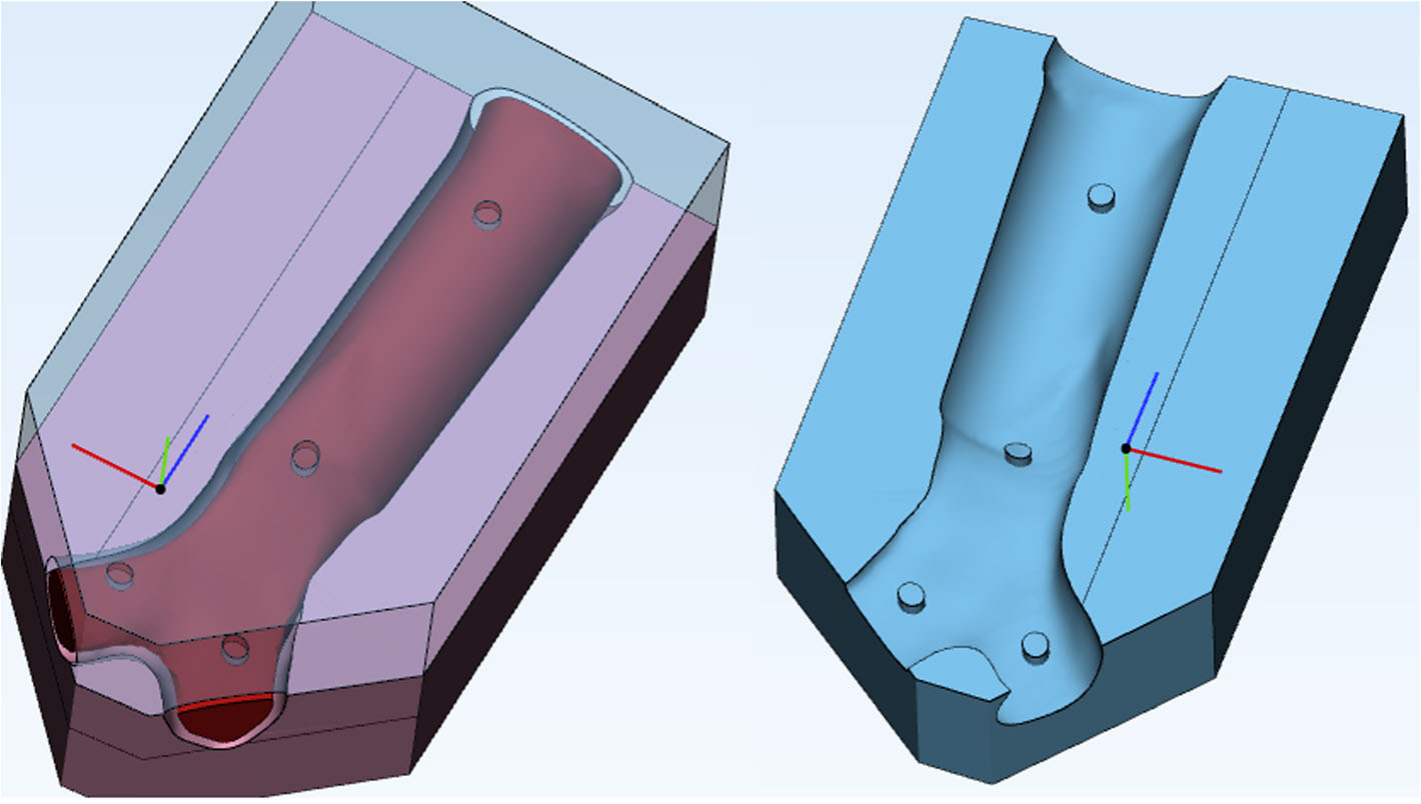
Final mold assembled (left) and with the upper part demonstrated separately. Note the support pins, which can be of variable diameter

Boolean subtraction of the inner part of the mold from the upper and lower external parts of the mold will ensure precise positioning of the inner part on the support pins, thus yielding a usable, aligned final cast. As a tradeoff for this alignment, several surface defects will be present on the stent, but are most likely to be of limited clinical significance (Fig. 42).

## Practical considerations of 3D printing software

In principle, the process of submitting a model to a 3D printer is highly variable, and ranges from placement of a model onto an external storage device which is then inserted into the printer to using a graphical user interface to submit a model to a printer connected to the computer directly or through a network. The end result, however, is the translation of the 3D geometry stored within STL or similar files into a set of printer instructions, such as movement paths for the printer head, print bed movement, and a range of instructions that are highly dependent on the printer used.

**Fig. 43:**
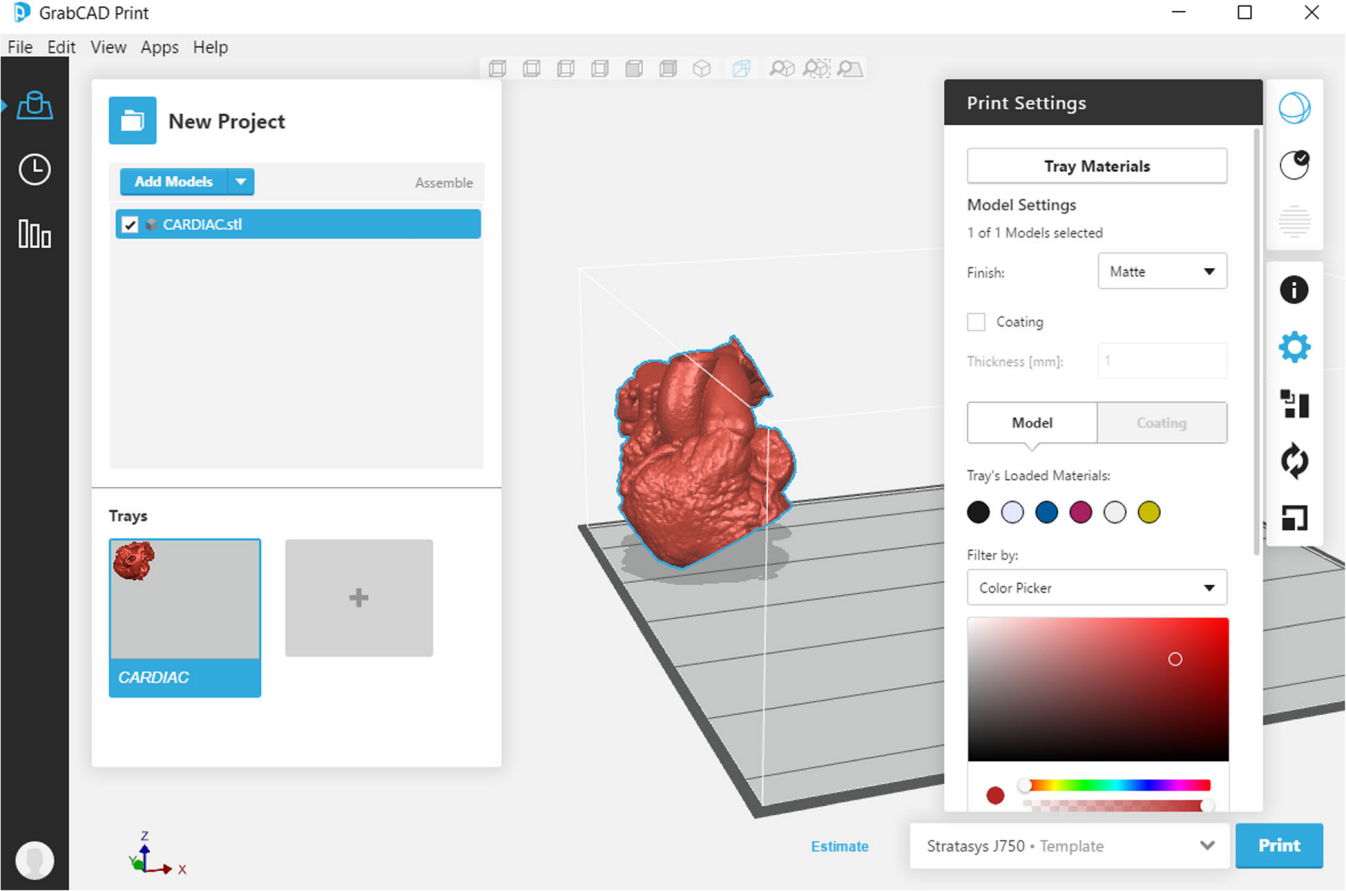
GrabCAD graphical user interface, with visualization of the printable 3D model on a dynamically rotatable tray that adheres to the dimensions of the selected printer. Associated print settings allow the selection of materials for individual models, as well as model placement and cost estimation

To demonstrate the practical aspects of 3D printing, this paper uses Stratasys GrabCAD, a standalone software and online printing service capable of handling a wide range of printers (Fig. 43).

The interface is intuitive and can be used to discuss submitting 3D models for printing in general. One typically imports the model as a standalone item or an assembly using any of a number of acceptable formats, including STL. In the case of the Pancoast tumor model generated here, one would import all parts as an assembly to ensure correct alignment in the complex multi-material model.

**Fig. 44:**
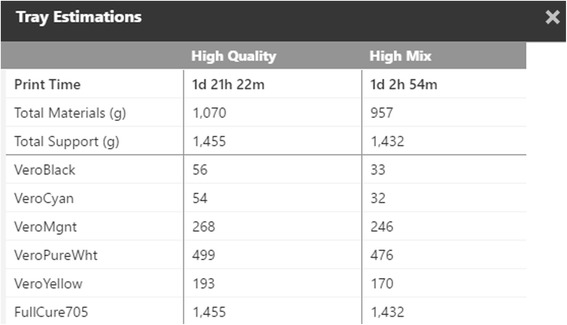
Results of dynamic cost estimates within the GrabCAD software. Typically, several printing options are provided -here, High Quality has a 14 μm resolution while High Mix results in 27 μm resolution prints

**Fig. 45:**
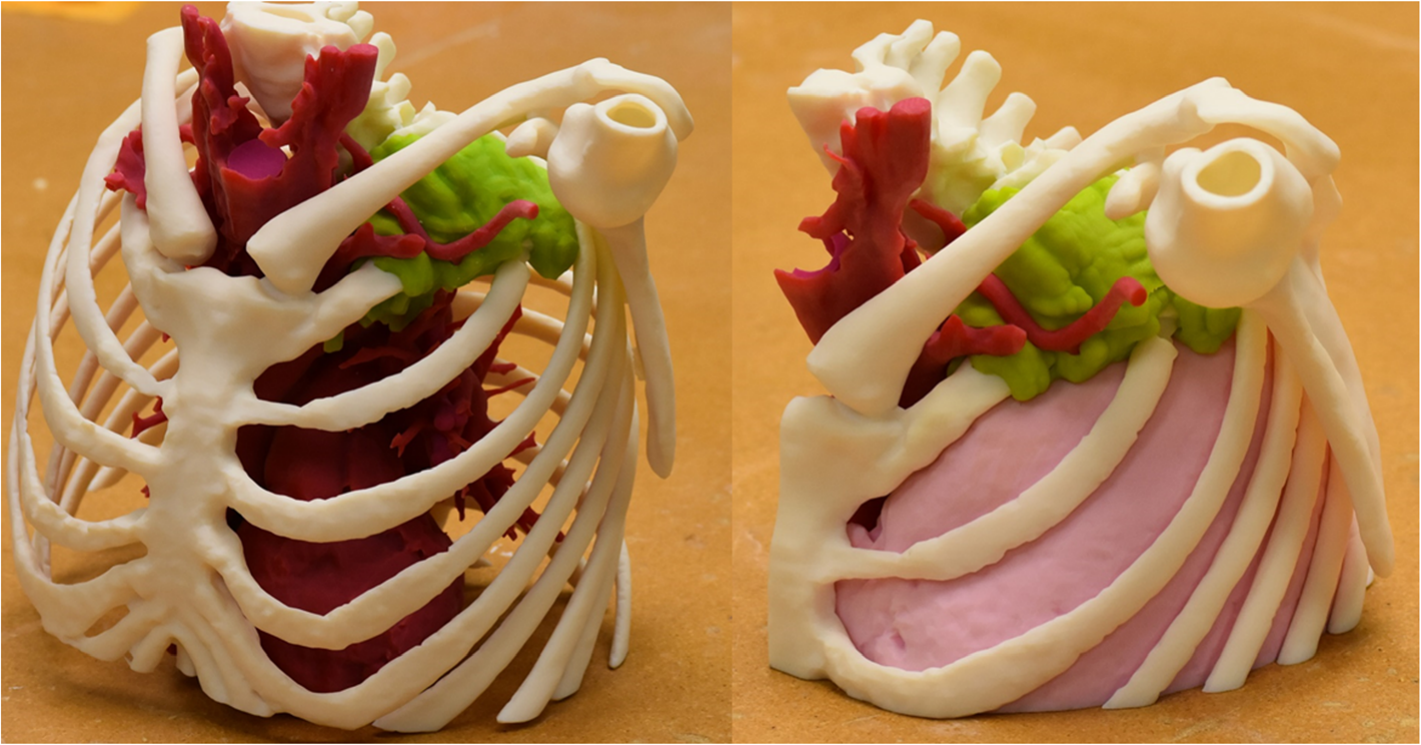
Left, a model of the entire bony and vascular thorax from the case presented here. Right, the left upper thorax segmented and further processed in this work. The tumor is shown in green, and closely abuts but does not involve the adjacent arterial vasculature

Automated or manual on-tray arrangement tools are then applied to minimize support material use, and upon satisfactory alignment, the material and time costs are estimated (Fig. 44). This is not always ideal following automated placement and manual adjustments are frequently required. Following satisfaction with this estimate, the print is initiated. Although this process often requires optimization for appropriate results, the overall experience across platforms has matured in the recent years tremendously and has been optimized to result in tremendously valuable medical models (Fig. 45).

## Conclusion

As applications of 3D printing in medicine continue to expand, familiarity with the basic software operations and tasks becomes increasingly important. This technical note presented at the 2017 annual meeting of the RSNA expands on our earlier teaching sessions to include segmentation and printing of a Pancoast tumor. Finally, the application of patient-specific airway stent design with CAD demonstrates some of the basic techniques involved in clinical applications of 3D printing.

## Additional file

Additional file 1: Segmentation. (MCS 92160 kb)

Additional file 2: Pancoast CAD. (MXP 14411 kb)

Additional file 3: Stent design. (MXP 2364 kb)

## Abbreviations

3D: Three dimensional
CAD: Computer-aided design
CT: Computed tomography
DICOM: Digital imaging and communications in medicine
FDA: United States Food and Drug Administration
HU: Hounsfield units
MRI: Magnetic resonance imaging
ROI: Region of interest
RSNA: Radiological Society of North America
STL: Standard tessellation language/Stereolithography
